# Vertical Cancer Transmission via Asexual Fragmentation and Associated Cancer Prevalence

**DOI:** 10.1111/eva.70111

**Published:** 2025-05-21

**Authors:** Jibeom Choi

**Affiliations:** ^1^ Department of Applied Mathematics Kyung Hee University Yongin Republic of Korea; ^2^ School of Computational Sciences Korea Institute for Advanced Study Seoul Republic of Korea

**Keywords:** binary fission, budding, cancer, fissiparity, primordial stem cell

## Abstract

While sexual reproduction is a general feature of animals, fissiparity and budding are relatively uncommon modes of asexual reproduction by which a fragment from a parent becomes an independent organism. Unlike unitary development, tumor cells can be included in the detached fragment destined to become offspring. Although fragmentation facilitates the vertical transmission of parental tumor cells to nascent progeny, this process requires significantly fewer cell replications than development from a zygote. The former is a risk factor for cancer, while the latter reduces oncogenic mutations during replication, indicating that two opposite effects of carcinogenesis are involved in fragmentation. If fragmentation can significantly reduce the number of cell replications for the development and a small portion of parental cancer is transmitted to the offspring during fragmentation, consecutive fragmentation across generations can gradually diminish the cancer risk of offspring, which I term fragmentational purging. On the other hand, consecutive fragmentation may aggravate the cancer risk of the progeny, a process of fragmentational accumulation. The model results imply that fragmentational purging does not necessarily guarantee the evolution of fragmentation, nor does fragmentational accumulation ensure its exclusion. Other relevant factors including juvenile susceptibility of sexual reproduction and loss of genetic diversity stemming from asexual reproduction can influence the selective advantage of fragmentation. Furthermore, owing to the common features of stemness and self‐renewal, the existence of pluripotent adult stem cells required for fragmentation could be coupled with elevated cancer risk. The model results across diverse parameters and the associated mathematical analyses highlight multifaceted evolutionary trajectories toward fragmentation. Further investigation of cancer‐suppression strategies that fragmentational animals employ could provide insights into regenerative medicine and cancer therapy.

## 1 Introduction

As an outcome of the major transition in the history of life, metazoan multicellularity is characterized by the dependency of constituent cells on the organism and the division of labor (differentiation). For instance, a human pancreatic beta cell (unless it is cancerous) is specialized for the secretion of insulin and cannot persist when detached from the body. The conventional understanding of metazoan obligate multicellularity is that germ cell lineages monopolize the capability to transmit their genetic materials to the subsequent generations, referred to as the Weissmann barrier (Solana 2013). Somatic cells including the cancer cells, on the other hand, eventually perish when the host organism is deceased except for few eccentric cells such as HeLa cells (Masters 2002). However, some types of cancer can be transmitted from organisms to organisms (Ujvari et al. 2016). In humans, maternal cancer can be vertically transmitted to infants during pregnancy (transplacental cancer transmission) (Tolar and Neglia 2003). Cancer can be horizontally transmitted via canine coitus or biting between Tasmanian devils (Ujvari et al. 2016; Pearse and Swift 2006; Belov 2011). Transmission of tumors via surgical organ transplantation has been reported (Kauffman et al. 2002).

**FIGURE 1:**
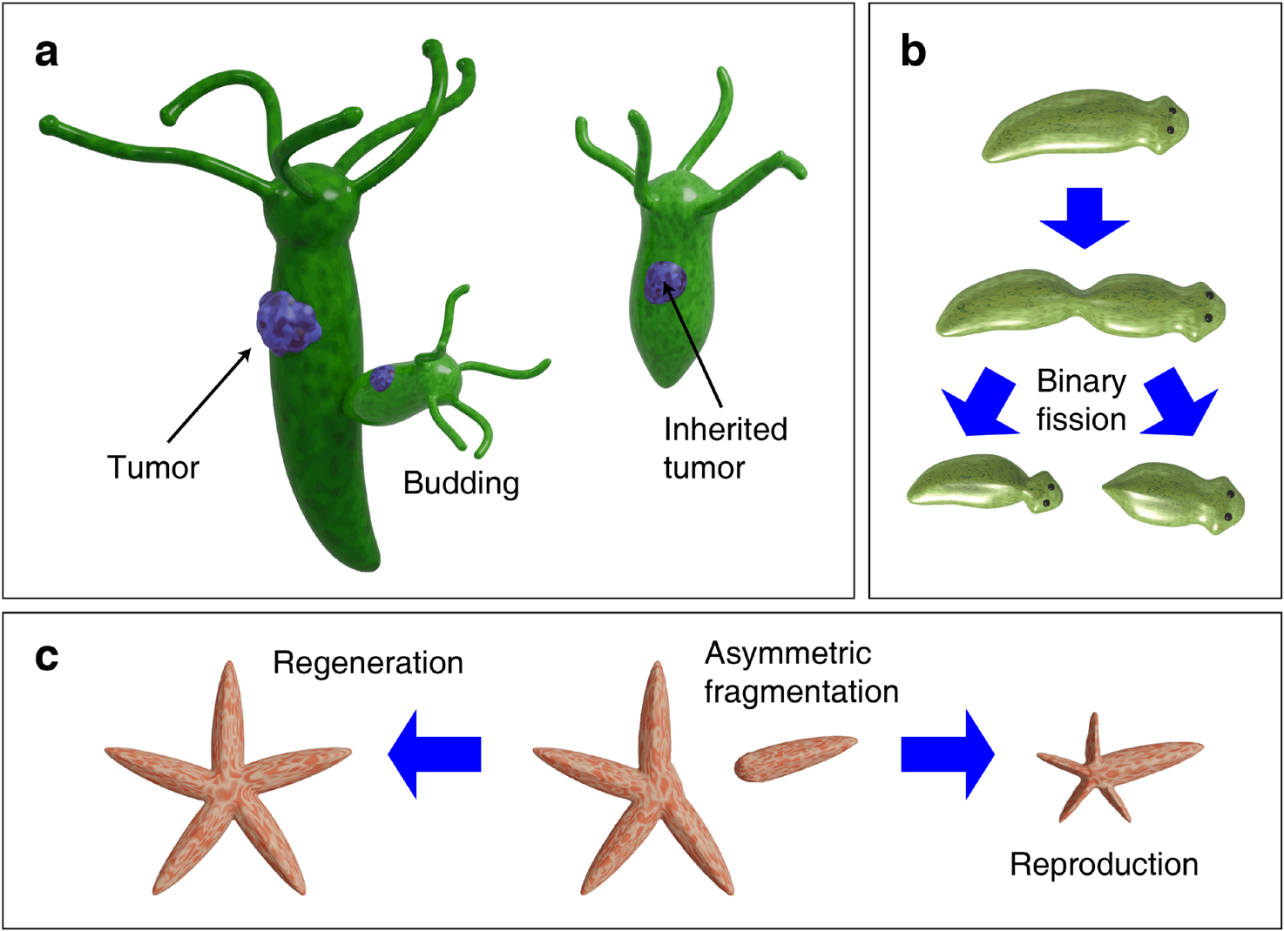
The modes of asexual fragmentation and possible mechanism of vertical cancer transmission. (a) The tumor in *Hydra* can be vertically transmitted to the progeny produced by budding (after Domazet‐Lošo et al. 2014). (b) Tumor cells in planaria performing transverse binary fission would be somatically transmitted to the subsequent generation. (c) Tumor cells in a detached arm of the starfish (fissiparity) will remain in the offspring.

The less attended mode of reproduction is fissiparity, by which an organism is divided into parts and each part becomes an independent organism. As the fragments are composed of multiple cells, the cancer could be included in fragments and transmitted to the subsequent generation. Similarly, cancer cells of the parental organism could be incorporated into the offspring produced by asexual budding. As an evident example, Domazet‐Lošo et al. (2014) experimentally confirmed that transplanted tumors of *Hydra* can be transmitted to the offspring produced by the budding (Figure 1a). A higher migration rate of the *Hydra* tumor cells would facilitate the transmission of the tumor cells to the budding polyp. Another experimental result suggests that *Hydra* tumor cells might be vertically and iteratively transmitted across multiple generations (Tissot et al. 2024). Analogous processes of vertical tumor transmission in species that reproduce by fissiparity and budding can be envisaged (Figure 1b,c). For brevity, let fragmentation refer to fissiparity and budding. In this study, fragmentational purging refers to the reduction of the cancer cells in the mature progeny generated by fragmentation compared to cancer cells of the parental organism. If fragmentation elevates the proportion of cancer cells in the mature progeny, it is labeled as fragmentational accumulation (Definition 1 in Appendix S1).

As evidenced by retinoblastoma and other hereditary cancer syndromes, oncogenic mutations in the germ cells can affect the cancer risk of the offspring (Knudson Jr 1971). However, cancer cells themselves cannot be passed on to the offspring by sexual reproduction given that cancerous germ cells cannot form a functional zygote. On the other hand, development from the aggregation of the cells rather than a single zygote can significantly reduce the required cell replication number for maturation—the evident risk factor of carcinogenesis (Tomasetti and Vogelstein 2015). Such contrasting effects of fragmentation on cancer susceptibility pose a conundrum of whether fragmentation elevates or suppresses the susceptibility.

Another feature of the fragmentational (fissiparous or budding) species is the possession of pluripotent primordial stem cells (priSCs) after maturation. Conceptualized by Solana (2013), self‐renewing priSCs have both gametic and somatic potential whose inclusion into the germline generalizes the germline continuity established from the Weismann barrier (Solana 2013). It is speculated that priSCs, like germ cells or multipotent stem cells, exhibit a germline multipotency program (GMP) such as *piwi* or *nanos* (Solana 2013; Juliano et al. 2010). Notable examples of adult primordial stem cells (henceforth, APSCs) are planarian neoblasts and *Hydra* I‐cells (interstitial stem cells), both of which can produce diverse types of cells. Considering the fragmentational capability of the planarian and *Hydra* combined with the multipotency of priSCs, it is tantalizing to expect that possession of the APSCs could be the prerequisite of fragmentation. GMP‐expressing APSCs that are comparable to neoblasts and I‐cells are found in other fragmentational animals including starfish (Fujita et al. 2023; Magalhães et al. 2023), annelids (Álvarez‐Campos et al. 2024), sponges (Juliano et al. 2010), acoels (Hulett et al. 2023), and sea anemones (Miramón‐Puértolas et al. 2024). There is reliable evidence that there are APSCs in coral (López‐Nandam et al. 2023). Furthermore, APSCs will confer high regenerative capability as revealed in fragmentational animals (Table S1).

Despite the evident advantages of the APSCs, the development of the APSCs would be a double‐edged sword because they share multiple traits with cancer stem cells. Both types of cells can self‐renew and produce diverse differentiated cells. Due to such commonalities, cancer stem cells may originate from stem cells (Shackleton et al. 2009). Consistent with this reasoning, planarian neoblasts and *Hydra* I‐cells are likely to be implicated in tumorigenesis (Domazet‐Lošo et al. 2014; Voura et al. 2017; Van Roten et al. 2018; Boutry et al. 2023). In other words, APSCs may require a smaller number of driver mutations to become cancerous as they are already equipped with stemness and self‐renewal capability. Furthermore, GMP may play a critical role in cancer stem cells (Garcia‐Borja et al. 2024).

Distinct attributes of fissiparous or budding organisms including APSCs and vertical transmission of cancer cells to the offspring must be pivotal factors for cancer susceptibility and the evolution of cancer suppression. Specifically, how would features associated with fragmentation including fragmentation frequency, regeneration capability, or juvenile vulnerability influence the optimal strategy of cancer suppression? To investigate the effect of asexual fragmentation on cancer evolution, I built a graph‐theoretical pedigree framework illustrating different modes of sexual and asexual reproduction. During the lifetime of an organism in the proposed model, different types of cells accumulate mutation and proliferate, influenced by the level of cancer‐suppression capability.

The theoretical formulation based on evolutionary principles and examination of cancer susceptibility across diverse model parameters demonstrates the conditions that favor asexual fragmentation and how they affect cancer prevalence. This may explain the production of efficient tumor‐suppressing substances in numerous fragmentational marine organisms that have possible medical applications (Macedo et al. 2021). Furthermore, this study provides evolutionary insight into why comprehensive regeneration is inhibited in animals composed of highly differentiated cells. To my knowledge, no study to date has mathematically investigated the effect of vertical cancer transmission by fragmentation on cancer prevalence across multiple generations.

In this model, the cancer of multicellular animals (Metazoa) was taken into account. The plants were not considered because plant tumors are generally less lethal and caused by infections rather than spontaneous mutations (Doonan and Sablowski 2010).

## 2 Model Formulations

### 2.1 Cell Types and Organism Composition

**FIGURE 2:**
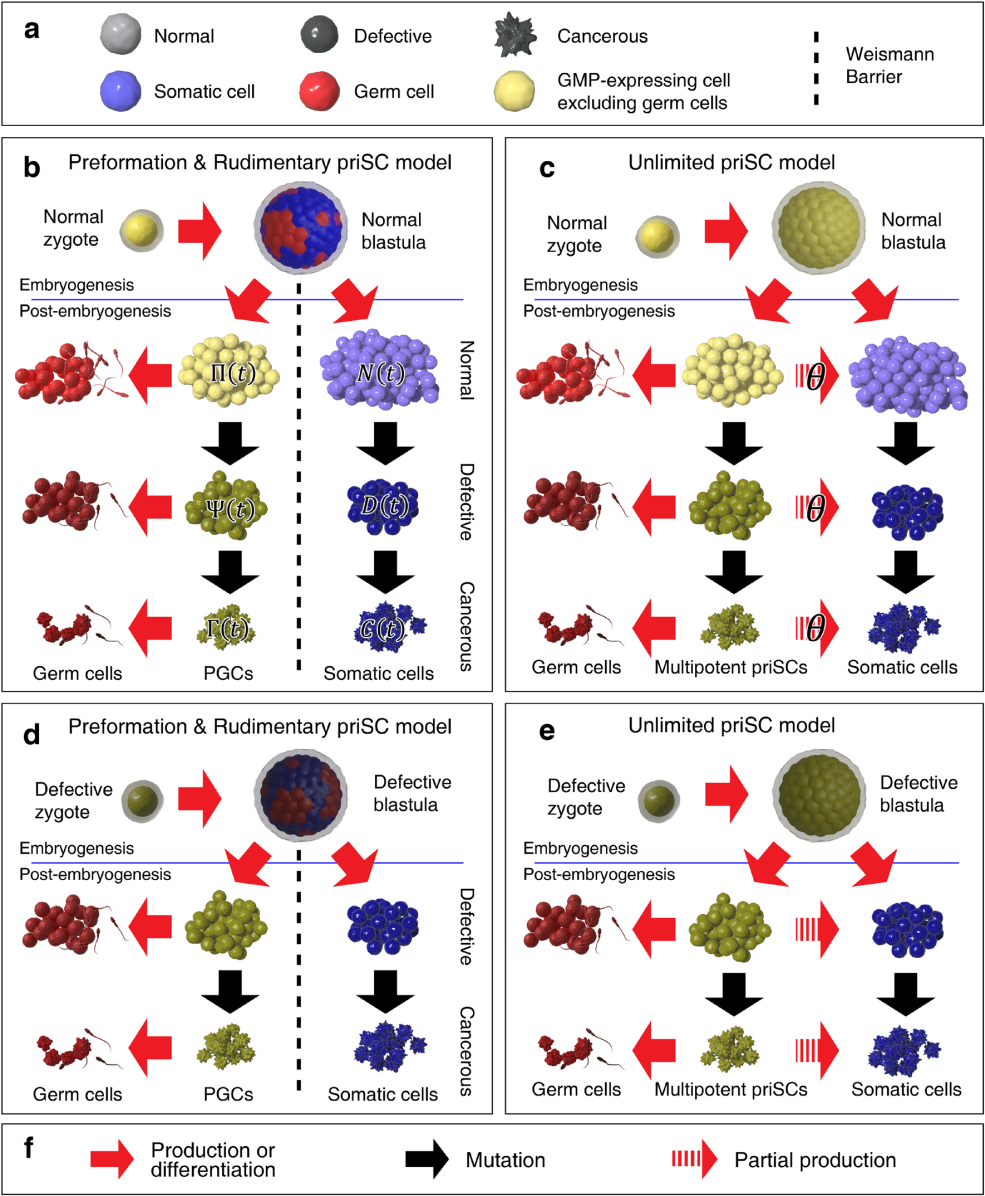
Growth and mutation diagram of unitary development (sexual reproduction) used in this study. (a) The brightness of a cell illustrates whether the cell is normal. Darker colors imply the existence of oncogenic mutations. Round‐shaped and bright cells are normal; round‐shaped and dark cells are defective; rumpled and dark cells are cancerous. Once embryogenesis is finished, the blue hue indicates somatic cells excluding APSCs. The red hue is allocated to germ cells (sperms and eggs). For an organism containing APSCs, yellow cells illustrate APSCs. When such cells are absent, yellow cells illustrate PGCs. Weismann barrier in this study follows the generalized framework proposed by Solana (2013). Under this perspective, APSCs are included in the germline as mutations in them can be directly transmitted to the progeny. (b) Under the preformation and rudimentary priSC model (Solana 2013), gametic and somatic fates are determined during embryogenesis. A normal zygote establishes a blastula composed of normal cells. The zygote is illustrated with a yellow hue considering their totipotency. Similarly, yellow cells in the blastula have gametic potential. After embryogenesis, PGCs (yellow cells) can produce germ cells, while mutations in somatic cells (blue cells) cannot be transmitted to the offspring. As replication proceeds, normal PGCs and somatic cells become defective, and defective cells become cancerous (black downward arrows). Normal, defective, cancerous PGCs are assumed to produce normal, defective, cancerous germ cells, respectively (red leftward arrows). (c) In this study, the possession of the multipotent APSCs is the prerequisite for the fragmentation. APSCs have both gametic and somatic potential. The composition of somatic cells under this model is partially influenced by APSC composition with the coefficient θ (Equation (10)). (d) Same as (b) when the zygote is a defective cell. This organism does not have any normal cells. (e) Same as (c) when the zygote is a defective cell. (f) The explanation of the arrows used in (b)–(e). APSC, adult primordial stem cell; GMP, germline multipotency program; PGC, primordial germ cell; priSC, primordial stem cell.

Based on the degree of oncogenic mutations in a cell, three levels of cell states were postulated in this model—normal cells, defective cells, and cancerous cells. Defective cells possess certain oncogenic mutations though they are yet to be cancerous. Mutations in normal cells turn them into defective cells. Additional mutations make defective cells cancerous. As revealed in Figure 2a, normal cells are illustrated with bright spherical morphology, while defective cells are in darker colors. Cancer cells exhibit rumpled surfaces.

Once embryonic development is finished, three categories of adult cells depending on their functions were postulated. Somatic cells including adult stem cells (except APSCs) or progenitor cells (except primordial germ cells) are illustrated with a blue hue. In other words, it is defined in this model that adult somatic cells cannot directly transmit their genetic material to the progeny via sexual reproduction (evolutionary bystanders). Normal cells that exhibit the GMP excluding the germ cells are described with a yellow hue. Under preformation model (Solana 2013), yellow cells after embryogenesis represent primordial germ cells (PGCs). On the other hand, yellow cells after embryogenesis represent PGCs and APSCs under unlimited priSC model (Solana 2013) such as neoblasts or I‐cells.

If developed from a normal zygote, all cells immediately after embryogenesis were assumed to be normal (Figure 2b,c). As replication proceeds, defective and cancerous cells arise. If developed from a defective zygote, all cells immediately after the embryogenesis were assumed to be defective (Figure 2d,e).

Let $\Pi \left(t\right)$, $\Psi \left(t\right)$, and $\Gamma \left(t\right)$ denote the proportions of the normal, defective, and cancerous PGCs/APSCs in an organism at t, respectively (Figure 2b). Here, t is the number of cell replications, postulating that t=0 immediately after embryogenesis or fragmentation. Alternatively, t may represent the age (the degree of senescence) of an organism given that the replication rate is constant. As these values represent the proportions, $\Pi \left(t\right)+\Psi \left(t\right)+\Gamma \left(t\right)=1$ regardless of t. Likewise, $N\left(t\right)$, $D\left(t\right)$, and $C\left(t\right)$ represent the proportions of normal, defective, and cancerous somatic cells.

Based on the two‐hit hypothesis for tumorigenesis (Knudson Jr 1971), it was postulated that the proportion of the normal PGCs/APSCs monotonically decreases as they transform into defective PGCs/APSCs or cancerous cells grow faster than normal cells. The defective PGCs/APSCs in turn transform into the cancerous PGCs/APSCs. As such, the following differential equations can be formulated.

(1) $$\frac{d\Pi \left(t\right)}{\mathrm{dt}}=-\mu_{1}\Pi \left(t\right)$$

(2) $$\begin{array}{c} \frac{d\Psi \left(t\right)}{\mathrm{dt}}=\mu_{1}\Pi \left(t\right)-\mu_{2}\Psi \left(t\right) \end{array}$$

(3) $$\begin{array}{c} \frac{d\Gamma \left(t\right)}{\mathrm{dt}}=\mu_{2}\Psi \left(t\right) \end{array}$$

where $\mu_{1}$ is the transition rate (determined by mutation rate and proliferation of each cell type) from normal to defective PGCs/APSCs, and $\mu_{2}$ is that from defective to cancerous PGCs/APSCs. The general solutions for these differential equations are

(4) $$\begin{array}{c} \Pi \left(t\right)=\Pi \left(0\right)\exp\left[-\mu_{1}t\right] \end{array}$$

(5) $$\begin{array}{c} \Psi \left(t\right)=\Psi \left(0\right)\exp\left[-\mu_{2}t\right]+\mu_{1}\Pi \left(0\right)\frac{\exp\left[-\mu_{1}t\right]-\exp\left[-\mu_{2}t\right]}{\mu_{2}-\mu_{1}} \end{array}$$

(6) $$\begin{array}{c} \Gamma \left(t\right)=1-\Psi \left(0\right)\exp\left[-\mu_{2}t\right]-\Pi \left(0\right)\frac{\mu_{2}\exp\left[-\mu_{1}t\right]-\mu_{1}\exp\left[-\mu_{2}t\right]}{\mu_{2}-\mu_{1}} \end{array}$$

The transition dynamics of the somatic cells have identical structures.

(7) $$\begin{array}{c} \frac{\mathrm{dN}\left(t\right)}{\mathrm{dt}}=-k_{1}N\left(t\right) \end{array}$$

(8) $$\begin{array}{c} \frac{\mathrm{dD}\left(t\right)}{\mathrm{dt}}=k_{1}N\left(t\right)-k_{2}D\left(t\right) \end{array}$$

(9) $$\begin{array}{c} \frac{\mathrm{dC}\left(t\right)}{\mathrm{dt}}=k_{2}D\left(t\right) \end{array}$$

where $k_{1}$ and $k_{2}$ are parameters for somatic mutations that correspond to $\mu_{1}$ and $\mu_{2}$.

Frequent oncogenic mutations will elevate the transition rates ($\mu_{1}$, $\mu_{2}$, $k_{1}$, $k_{2}$). Consequently, the immunity and the functionality of the tumor‐suppression mechanism should influence the transition rates. The parameter I in this study represents such a combined cancer‐suppression capability. Although the oncogenic mutation is absent, the higher growth rate of cancer cells can make transition rates positive as the higher proportion of cancer cells results in a reduced proportion of normal and defective cells.

**FIGURE 3:**
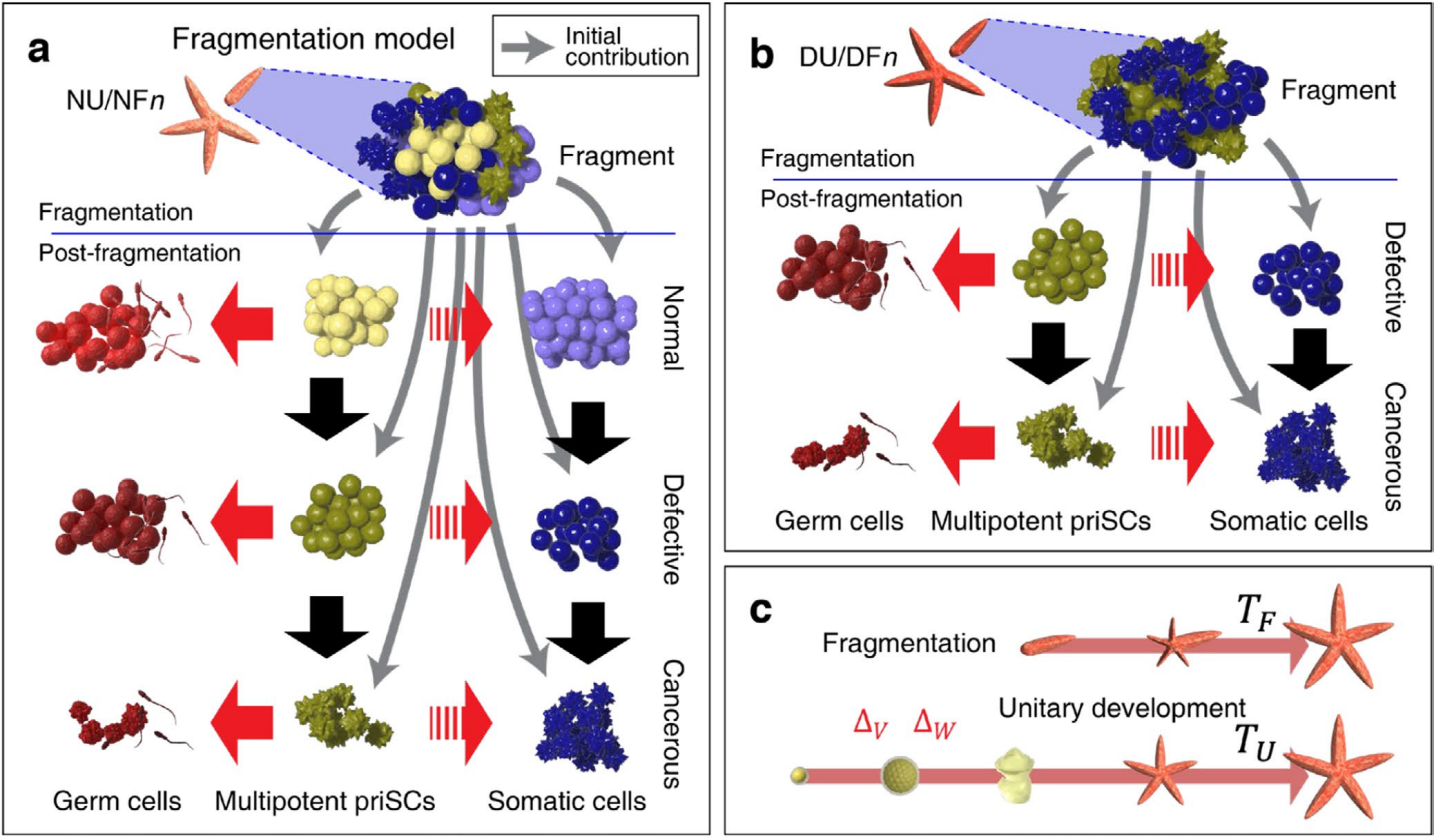
Growth and mutation diagram of fragmentation used in this study. (a) A detached fragment (or bud) from a NU or NF*n* organism is composed of diverse types of cells with different oncogenicity. Following the detachment, the cells in the fragment replicate during which mutation arises. Fragmentation postulates the existence of adult primordial stem cells (APSCs) such as planarian neoblasts and *Hydra* I‐cells. (b) If the detached fragment originates from a DU or DF*n* organism, no normal cell is included in the fragment. (c) The number of cell replications to reach maturity would be different for unitary development and fragmentation. For unitary development, a single cell needs to replicate to establish a mature organism, hence the required number of the replication would be larger than that of the fragmentation ($T_{U}>T_{F}$). In addition, an organism produced via unitary development needs to go through the juvenile period which is vulnerable to predation and starvation. The survival risk and associated fitness reduction of juvenility are reflected in $\Delta_{V}$ and $\Delta_{W}$. APSC, adult primordial stem cell; priSC, primordial stem cell.

For an organism developed from a normal zygote, $\left[\Pi \left(0\right),\Psi \left(0\right),\Gamma \left(0\right)\right]=\left[N\left(0\right),D\left(0\right),C\left(0\right)\right]=\left[1,0,0\right]$. If developed from a defective zygote, $\left[\Pi \left(0\right),\Psi \left(0\right),\Gamma \left(0\right)\right]=\left[N\left(0\right),D\left(0\right),C\left(0\right)\right]=\left[0,1,0\right]$. The initial proportion of the PGCs/APSCs and somatic cells of an organism developed via fissiparity or budding is determined by the composition of the parental organism (Figure 3a,b), which will be explained later.

A single zygote needs to repeatedly replicate to reach the cell number of the mature organism. In contrast, a fragment that contains multiple cells needs to replicate fewer times to reach the cell number of maturity. Accordingly, the time or the number of replications to reach maturity would be larger for unitary development compared to those of fragmentation (Figure 3c). Let $T_{U}$ and $T_{F}$ denote the number of replications (or equivalent to the time given that the replication rate is constant) required to reach maturity by unitary development and fragmentation, respectively. It was assumed in the model that $T_{U}>T_{F}$. Refer to the Section 3 for the simplified mathematical analysis.

**FIGURE 4:**
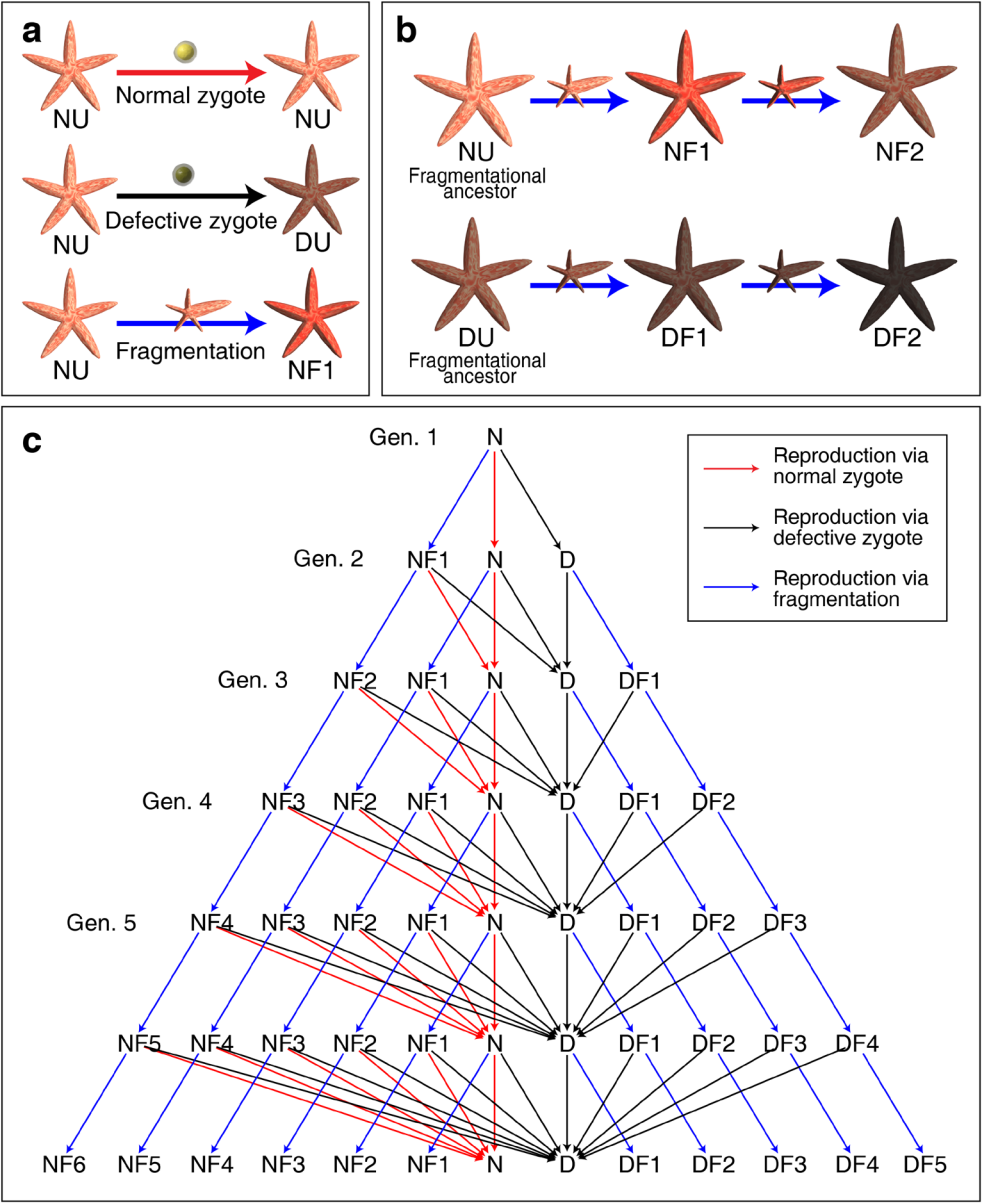
The modes of reproduction and population dynamics used in this study. (a) An NU organism can produce a normal zygote which will later become an NU offspring. An NU organism can also produce a defective zygote, becoming a DU offspring. If a progeny is established from a fragment detached from the NU parent, the progeny becomes an NF1 organism. (b) NF1 organism can produce NF2 offspring by fragmentation. The NU organism is the fragmentational ancestor of NF1, NF2, and other NF*n* organisms. Likewise, the DU organism is the fragmentational ancestor of DF*n* organisms where *n* refers to the number of consecutive fragmentations across generations. (c) The pedigree of the model. At the first generation (Gen. 1), all population was assumed to be composed of NU organisms. Owing to spatial limitations, N and D refer to NU and DU organisms, respectively. NU organisms can produce NU, DU, and NF1 organisms in the subsequent generation (Gen. 2). NF*i* and DF*i* organisms can produce NF(*i* + 1) and DF(*i* + 1) organisms, respectively, by fragmentation. In addition, NF*i* organisms, by sexual reproduction, can produce NU and DU organisms, while DF*i* organisms can only produce DU organisms by sexual reproduction. Based on the subpopulation size and fitness of each type, the population composition of the subsequent generation can be estimated. Red and black arrows indicate the unitary development via normal and defective zygotes, respectively. The blue arrow corresponds to the fragmentation.

As all metazoans can reproduce by unitary development (sexual reproduction or parthenogenesis) (Queller 2000; Grosberg and Strathmann 1998), the same argument was set in this model. Considering that parthenogenesis without sexual reproduction is not a common mode of reproduction in nature (Vakhrusheva et al. 2020), unitary development solely refers to the outcome of sexual reproduction in this study. A new organism can be established from a normal or defective zygote (Figure 4a). Let type NU denote the organisms originating from a normal zygote. Type DU refers to the organism developed from a defective zygote. All cells of DU organisms are either defective or cancerous.

Let $\Pi^{V}$, $\Psi^{V}$ and, $\Gamma^{V}$ denote the proportion of normal, defective, and cancerous PGCs/APSCs of NU organisms at maturity, respectively. Note that the superscript V is not the numeric value representing the power. Rather, it is a categorical label representing the NU type. Specifically, $\Pi^{V}=\Pi \left(T_{U}\right)$ and $\Psi^{V}=\Psi \left(T_{U}\right)$ with $\Pi \left(0\right)=1$ (Equations (4), (5), (6)). In addition, $\Gamma^{V}=1-\Pi^{V}-\Psi^{V}$. For DU organisms, $\Pi^{W}=0$ and $\Psi^{W}=\Psi \left(T_{U}\right)$ with $\Psi \left(0\right)=1$. Similarly, let $N^{V}$ and $D^{V}$ denote the proportion of normal and defective somatic cells of NU organisms at maturity. Under the preformation model lacking APSCs, $N^{V}=N\left(T_{U}\right)$ and $D^{V}=D\left(T_{U}\right)$ with $N\left(0\right)=1$.

Let θ denote the proliferability of APSCs (0≤θ<1), which influences the production of somatic cells, oncogenicity, and regenerative capability. If APSCs that can generate the somatic cells are present, the composition of the somatic cells at maturity is influenced by the parameter θ (Figure 2c,e).

(10) $$\begin{array}{c} \left[\begin{array}{c} N^{V}\\ D^{V}\\ 1-N^{V}-D^{V} \end{array}\right]=\theta \left[\begin{array}{c} \Pi^{V}\\ \Psi^{V}\\ 1-\Pi^{V}-\Psi^{V} \end{array}\right]+\left(1-\theta \right)\left[\begin{array}{c} N\left(T_{U}\right)\\ D\left(T_{U}\right)\\ 1-N\left(T_{U}\right)-D\left(T_{U}\right) \end{array}\right] \end{array}$$

which indicates that the composition of APSCs ($\Pi^{V}$, $\Psi^{V}$, $\Gamma^{V}$) affects the composition of somatic cells at maturity ($N^{V}$, $D^{V}$). Using such relations, the somatic cell composition of DU organisms under an unlimited priSC model can be similarly described.

Higher APSC proliferability was assumed to confer higher regenerative capability that alleviates the damage of the physical trauma. As a trade‐off, higher APSC proliferability is associated with higher transition rates of APSCs ($\mu_{1}$, $\mu_{2}$) based on the fact that proliferation is linked to tumorigenesis (Jacqueline et al. 2017; Ratajczak et al. 2018; Cho et al. 2024). Consequently, the transition rates are primarily determined by the cancer‐suppression capability (0<I<1) combined with APSC proliferability and biased replication fidelity (s).

(11) $$\begin{array}{c} k_{1}=\mathrm{s\eta}\left(I\right) \end{array}$$

(12) $$\begin{array}{c} \mu_{1}=\left(1+\theta \right)\left(2-s\right)\eta \left(I\right) \end{array}$$

Here, $\eta \left(I\right)$ is the baseline transition rate determined by the cancer‐suppression capability I. As a higher cancer‐suppression capability reduces mutation rates, $\eta \left(I\right)$ is monotonically decreasing against I. Refer to Equation (S5) for its details. While I is the major contributor to cancer suppression, there could be supplementary APSC‐specific or somatic‐cell‐specific mechanisms of tumor suppression such as differential gene expression or allocation of quiescent subpopulation (Van Roten et al. 2018; Cheung and Rando 2013; Mihaylova et al. 2018). s and $\left(2-s\right)$ represent such disproportionate replication fidelity in PGCs/APSCs and somatic cells. If s=0.5, for instance, the mutation in somatic cells is suppressed at the cost of elevated mutations in PGCs/APSCs. In this model, five values of s were evaluated: 0.5, 0.75, 1, 1.25, 1.5. Furthermore, higher APSC proliferability elevates mutation risk (Jacqueline et al. 2017; Ratajczak et al. 2018; Cho et al. 2024), implemented in the term $\left(1+\theta \right)$ of Equation (12). For organisms that lack APSCs, θ=0.

Stemming from genomic instability or haploinsufficiency, inactivation of an allele of tumor‐suppression genes can facilitate the subsequent genetic alterations required for carcinogenesis (Smilenov 2006; Konishi et al. 2011). As such, transition rates from defective to cancerous cells were assumed to be higher than those from normal to defective cells. Specifically, $k_{2}=1.5k_{1}$ and $\mu_{2}=1.5\mu_{1}$.

Regardless of the organism types, let $N_{P}$, $D_{P}$, and $C_{P}$ denote proportions of the normal, defective, and cancerous somatic cells at maturity, respectively. Likewise, $\Pi_{P}$, $\Psi_{P}$, and $\Gamma_{P}$ are the proportions of the normal, defective, and cancerous PGCs/APSCs at maturity, respectively. The inherited composition of the progeny produced by fragmentation at birth ($\Pi_{F},\Psi_{F},\Gamma_{F}$) is

(13) $$\begin{array}{c} \left[\begin{array}{c} \Pi_{F}\\ \Psi_{F}\\ \Gamma_{F} \end{array}\right]=\frac{1}{f_{1}\Pi_{P}+f_{2}\Psi_{P}+f_{3}\Gamma_{P}}\left[\begin{array}{c} f_{1}\Pi_{P}\\ f_{2}\Psi_{P}\\ f_{3}\Gamma_{P} \end{array}\right] \end{array}$$

Composition of somatic cells ($N_{F}$, $D_{F}$, $C_{F}$) after fragmentation follows the identical relation.

(14) $$\begin{array}{c} \left[\begin{array}{c} N_{F}\\ D_{F}\\ C_{F} \end{array}\right]=\frac{1}{f_{1}N_{P}+f_{2}D_{P}+f_{3}C_{P}}\left[\begin{array}{c} f_{1}N_{P}\\ f_{2}D_{P}\\ f_{3}C_{P} \end{array}\right] \end{array}$$

Three nonnegative parameters $f_{1}$, $f_{2}$, $f_{3}$ represent the vertical transmission degree of normal, defective, and cancerous cells. If cancerous cells are highly mobile so that they are readily included in the dividing fragment as in the case of *Pelmatohydra robusta* (Domazet‐Lošo et al. 2014), $f_{3}$ would not be significantly smaller than $f_{1}$.

The fitness of each organism (ω) is determined by the composition of the somatic cells, cancer‐suppression capability, and the cost of the damage (physical trauma such as injury or predator attacks).

(15) $$\begin{array}{c} \omega =\omega_{0}+\alpha_{N}N_{P}^{\varepsilon }+\alpha_{D}D_{P}^{\varepsilon }+\alpha_{C}C_{P}^{\varepsilon }-\chi \left(\theta \right)-g\left(I\right) \end{array}$$

where $\omega_{0}$ is the baseline fitness, $\chi \left(\theta \right)$ is the effect of external damage (trauma), and $g\left(I\right)$ is the cost of sustaining the cancer‐suppression capability. $\alpha_{N}$, $\alpha_{D}$, and $\alpha_{C}$ are coefficients that determine how cell composition influences fitness. As a higher proportion of normal cells and a lower proportion of cancer cells will elevate fitness, it was postulated that $\alpha_{N}\geq \alpha_{D}\geq \alpha_{C}$. The power ε (exponent for $N_{P}^{\varepsilon }={\left(N_{P}\right)}^{\varepsilon }$) applied to $N_{P}$, $D_{P}$, and $C_{P}$ demonstrates the aggressive and lethality of cancer. As $N_{P}$, $D_{P}$, and $C_{P}$ are proportional values such that $N_{P}+D_{P}+C_{P}=1$ and $0\leq N_{P},D_{P},C_{P}\leq 1$, the increase in $C_{P}$ results in a corresponding decrease in $N_{P}$ or $D_{P}$. As a simple example, compare two cases of somatic composition $x_{1}=\left[1,0,0\right]$ and $x_{2}=\left[0.9,0,0.1\right]$. Here, $x_{1}$ depicts an organism purely composed of normal somatic cells. If 10% of the normal cells become cancerous, it is equivalent to $x_{2}$. Let us calculate $\alpha_{N}N_{P}^{\varepsilon }+\alpha_{D}D_{P}^{\varepsilon }+\alpha_{C}C_{P}^{\varepsilon }$ for $\alpha_{N}=1$ and $\alpha_{C}=0$. If ε=1, this value is 1 for $x_{1}$, and 0.9 for $x_{2}$. If ε=2, on the other hand, the value is still 1 for $x_{1}$, but it becomes 0.81 (= 0.9^2^) for $x_{2}$. In general, values of ε higher than 1 are associated with aggressiveness or fatality of cancer as a slight increase in $C_{P}$ leads to a significant drop in total fitness. In simulation models, I assigned the values of ε from 1 to 2 depending on cancer fatality (Table S2) where higher ε implies that cancer is life‐threatening to that species.

$\chi \left(\theta \right)$ is the fitness reduction due to the external damage that is dependent on the APSC proliferability (θ). Specifically,

(16) $$\begin{array}{c} \chi \left(\theta \right)=\tau \delta {\left(1-\theta \right)}^{2} \end{array}$$

where τ is the frequency of the external damage and δ is the severity of the damage in each traumatic event. Higher APSC proliferability reduces the expected damage owing to the high regenerative capability, reflected by ${\left(1-\theta \right)}^{2}$.

As in the case of the tumor‐suppression gene *p53*, higher cancer‐suppression capability enhances the replication fidelity while overall fitness is accordingly reduced (Jacqueline et al. 2017; Tyner et al. 2002). Hence, $g\left(I\right)=c_{0}I^{2}$ for a specific nonnegative coefficient $c_{0}$.

### 2.2 Modes of Reproduction and Population Dynamics

The label NF1 indicates that an organism is reproduced by the fragmentation of an NU organism (Figure 4b). If an NF1 organism produces an offspring by fragmentation, the offspring is NF2. In general, the NF*n* organism produces NF(*n* + 1) organisms by fragmentation. For NF*n* organisms, the fragmentational ancestor is an NU organism. In other words, fragmentational ancestor is defined as the most ancestral organism in the pedigree that initiated the repeated fragmentation. Here, *n* denotes the number of consecutive fragmentations across generations. DF*n* organisms can be analogously defined whose fragmentational ancestor is a DU organism.

NF*n* organisms can produce normal or defective zygotes. Hence, the NF*n* organisms can produce NU or DU organisms by sexual reproduction (unitary development). In contrast, it was assumed that DF*n* organisms can only produce defective zygotes as they do not possess normal cells. Thus, all progeny of DF*n* organisms produced by unitary development are DU organisms.

In each reproductive event of an organism, ϕ is the probability that the organism reproduces by fragmentation. Accordingly, 1−ϕ is the probability to reproduce sexually. Let $V_{j}$ and $W_{j}$ denote the subpopulation size of the NU and DU organisms at the *j*‐th generation, respectively. Likewise, $Z_{i,j}$ and $Q_{i,j}$ are the subpopulation size of NF*i* and DF*i* organisms at the *j*‐th generation.

As previously explained, the proportions of the normal, defective, and cancerous PGCs/APSCs of NU organisms at maturity are $\Pi^{V}$, $\Psi^{V}$, and $\Gamma^{V}$. It was assumed that cancerous germ cells cannot develop into an organism by unitary development. In each reproduction by unitary development, a normal gamete will be more likely to succeed in reproduction compared to a defective or cancerous gamete. Therefore, the contribution of the NU organisms in the *j*‐th generation to the subpopulation size of the NU organisms in the (*j* + 1)‐th generation is proportional to

(17) $$\begin{array}{c} \left(1-\phi \right)\frac{r_{1}\Pi^{V}}{r_{1}\Pi^{V}+r_{2}\Psi^{V}+r_{3}\Gamma^{V}}V_{j}\omega^{V} \end{array}$$

where $\omega^{V}$ is the fitness (reproductive success) of an NU organism calculated by Equation (15). Again, the superscript V is not a numeric value representing the power: it is the label for NU organisms. $r_{1}$, $r_{2}$, $r_{3}$ reflect the differential success rates in the fertilization of normal, defective, and cancerous germ cells.

For NF*i* organisms, their contribution to the subpopulation size of NU organisms in the subsequent (*j* + 1)‐th generation is proportional to

(18) $$\begin{array}{c} \left(1-\phi \right)\frac{r_{1}\Pi_{i}^{Z}}{r_{1}\Pi_{i}^{Z}+r_{2}\Psi_{i}^{Z}+r_{3}\Gamma_{i}^{Z}}H_{j}Z_{i,j}\omega_{i}^{Z} \end{array}$$

where $\Pi_{i}^{Z}$, $\Psi_{i}^{Z}$, and $\Gamma_{i}^{Z}$ are the proportions of normal, defective, and cancerous PGCs/APSCs of mature NF*i* organisms, respectively. Here, as in the case of $\Pi^{V}$, the superscript Z is assigned to NF*i* organisms. $\omega_{i}^{Z}$ is the fitness of an NF*i* organism, which is multiplied by $H_{j}$, the effect of genetic diversity (refer to Equation (22)). Consequently, the untransformed subpopulation size of NU organisms in the (*j* + 1)‐th generation is

(19) $$\begin{array}{c} V_{j+1}^{*}=\Delta_{V}\left(1-\phi \right)\left[V_{j}\omega^{V}U_{1}^{V}+H_{j}\sum_{i=1}^{j-1}Z_{i,j}\omega_{i}^{Z}U_{1,i}^{Z}\right] \end{array}$$

where

(20) $$\begin{array}{c} U_{1}^{V}=\frac{r_{1}\Pi^{V}}{r_{1}\Pi^{V}+r_{2}\Psi^{V}+r_{3}\Gamma^{V}} \end{array}$$

(21) $$\begin{array}{c} U_{1,i}^{Z}=\frac{r_{1}\Pi_{i}^{Z}}{r_{1}\Pi_{i}^{Z}+r_{2}\Psi_{i}^{Z}+r_{3}\Gamma_{i}^{Z}} \end{array}$$

$U_{1}^{V}$ demostrates the probability that an NU organism produces a normal zygote. $U_{1,i}^{Z}$ is the same as $U_{1}^{V}$ for NF*i* organisms. 1−ϕ reflects the probability that an organism attempts to reproduce by unitary development. $\Delta_{V}$ is the survival probability of the NU organisms' juvenile period ($0<\Delta_{V}<1$) (Figure 3c). For instance, echinoderm larvae would be exposed to the risk of predation and death due to their small size and immature mobility. $\Delta_{W}$ is the same as $\Delta_{V}$ for DU organisms. Organisms produced by the fragmentation do not experience such juvenile susceptibility in this model. If a cancerous germ cell succeeds in fertilization, the embryo cannot develop. Note that DU and DF*i* organisms cannot produce NU offspring because they do not possess normal germ cells.

The fragmentation produces clonal offspring, and the existence of multiple clonal organisms will make them susceptible to infectious disease and cancer (Thomas et al. 2019). Let $H_{j}$ denote the coefficient that describes the fitness reduction due to the loss of genetic diversity. Given that $\rho_{j}$ is the proportion of the NF*i* and DF*i* organisms at the *j*‐th generation,

(22) $$\begin{array}{c} H_{j}=b_{0}+\left(1-b_{0}\right){\left(1-\rho_{j}\right)}^{\frac{1}{4}} \end{array}$$

for $0<b_{0}<1$. The concavity of $H_{j}$ against $\rho_{j}$ explains that a smaller proportion of clones in the population has a negligible effect on population fitness. However, a higher proportion of clones drastically reduces the fitness of NF*i* and DF*i* organisms ($0\leq H_{j}\leq 1$). Lower values of $b_{0}$ are associated with the pronounced fitness reduction stemming from the genetic homogeneity (Figure S1).

The actual subpopulation size of the NU organisms at the subsequent generation ($V_{j+1}$) should consider the effect of the fluctuations in total population on the untransformed population size ($V_{j+1}^{*}$) which will be explained later.

All organisms in a generation are expected to possess defective germ cells. Hence, they can produce DU organisms by unitary development. By the same reasoning as $V_{j+1}^{*}$ was deduced,

(23) $$\begin{array}{c} W_{j+1}^{*}=\Delta_{W}\left(1-\phi \right)\left[V_{j}\omega^{V}U_{2}^{V}+W_{j}\omega^{W}U_{2}^{W}+H_{j}\sum_{i=1}^{j-1}Z_{i,j}\omega_{i}^{Z}U_{2,i}^{Z}+H_{j}\sum_{i=1}^{j-2}Q_{i,j}\omega_{i}^{Q}U_{2,i}^{Q}\right] \end{array}$$

where the asterisk implies that it is an untransformed subpopulation size. The detailed derivation processes are provided in Appendix S1.

NF1 organisms in the (*j* + 1)‐th generation are produced by NU organisms in the *j*‐th generation by asexual fragmentation (Figure 4).

(24) $$\begin{array}{c} Z_{1,j+1}^{*}=\phi V_{j}\omega^{V} \end{array}$$

Similarly, NF(*i* + 1) organisms in the (*j* + 1)‐th generation are produced by NF*i* organisms in the *j*‐th generation by asexual fragmentation.

(25) $$\begin{array}{c} Z_{i+1,j+1}^{*}=\phi H_{j}Z_{i,j}\omega_{i}^{Z} \end{array}$$

Likewise, DF*i* organisms satisfy the following equations.

(26) $$\begin{array}{c} Q_{1,j+1}^{*}=\phi W_{j}\omega^{W} \end{array}$$

(27) $$\begin{array}{c} Q_{i+1,j+1}^{*}=\phi H_{j}Q_{i,j}\omega_{i}^{Q} \end{array}$$

By the aforementioned relations, the total untransformed population size of (*j* + 1)‐th generation is determined.

(28) $$\begin{array}{c} P_{j+1}^{*}=V_{j+1}^{*}+W_{j+1}^{*}+\sum_{i=1}^{j}Z_{i,j+1}^{*}+\sum_{i=1}^{j-1}Q_{i,j+1}^{*} \end{array}$$

Considering the environmental capacity and to avoid the abrupt change in population size, it was assumed that the ratio of population size at the (*j* + 1)‐th and *j*‐th generation is bounded between 0.9391 to 1.08. These bounds are determined by the specific logistic function (Θ, Equation (S7)) designed to prevent rapid fluctuations of populations (Figure S2). The actual subpopulation size at the (*j* + 1)‐th generation was calculated by multiplying the untransformed subpopulation size by $\Theta \left(P_{j+1}^{*}/P_{j}\right)$. For example, $V_{j+1}=\Theta \left(P_{j+1}^{*}/P_{j}\right)V_{j+1}^{*}$ and $W_{j+1}=\Theta \left(P_{j+1}^{*}/P_{j}\right)W_{j+1}^{*}$. The sum of the individual organisms' fitness at the 30th generation was used as the optimization index of the reproduction strategy. The variables are explained in Table 1.

**TABLE 1:** The explanation of the major variables and abbreviations used in this study.

Variable or abbreviation	Description
priSC	Primordial stem cells (e.g., planarian neoblasts, *Hydra* I‐cells, 4d lineage of annelids and mollusks, the inner cell mass of mammalian embryo (Solana 2013))
APSC	Adult primordial stem cell
PGC	Primordial germ cell
GMP	Germline multipotency program
$\Pi \left(t\right)$, $\Psi \left(t\right)$, $\Gamma \left(t\right)$	The proportions of normal, defective, and cancerous PGCs/APSCs of an organism after t times of cell replications
$N\left(t\right)$, $D\left(t\right)$, $C\left(t\right)$	The proportions of normal, defective, and cancerous somatic cells of an organism after t times of cell replications
$\mu_{1}$, $\mu_{2}$, $k_{1}$, $k_{2}$	The transition rates that determine the transformation of normal cells into defective cells ($\mu_{1}$, $k_{1}$) and the transformation of defective cells into cancerous cells ($\mu_{2}$, $k_{2}$). $\mu_{1}$, $\mu_{2}$ represent the transition rates of PGCs/APSCs, and $k_{1}$, $k_{2}$ represent the transition rates of somatic cells. In addition to mutation rates, the differential growth rate of each cell type can affect the transition rates
ϕ	The probability that the organism reproduces by fragmentation in each reproductive attempt
$V_{j}$, $W_{j}$	The subpopulation sizes of the NU and DU organisms at the *j*‐th generation, respectively
$Z_{i,j}$, $Q_{i,j}$	The subpopulation sizes of NF*i* and DF*i* organisms at the *j*‐th generation
$V_{j}^{*}$, $W_{j}^{*}$, $Z_{i,j}^{*}$, $Q_{i,j}^{*}$	The untransformed subpopulation sizes of $V_{j}$, $W_{j}$, $Z_{i,j}$, $Q_{i,j}$, respectively. Applying the effect of total population fluctuation (Equation (S7)) determines the actual subpopulation size
$\omega^{V}$, $\omega^{W}$, $\omega_{i}^{Z}$, $\omega_{i}^{Q}$	The fitness of NU, DU, NF*i*, DF*i* organisms, respectively. Here, the superscripts are not numbers, but labels
θ	The proliferation index of APSCs which influences the production of somatic cells, cancer risk, and regenerative capability
ε	The parameter (power) that determines the fatality of cancer. A small increase in cancer proportion results in significant fitness reduction if ε is high
$T_{U}$, $T_{F}$	The numbers of cell replications required to obtain maturity for unitary development and fragmentation, respectively
$\Delta_{V}$, $\Delta_{W}$	The survival rates of NU and DU juvenile organisms developed from the zygote, respectively
$f_{1}$, $f_{2}$, $f_{3}$	The degrees of vertical transmission via fragmentation for normal, defective, and cancerous cells, respectively
$r_{1}$, $r_{2}$	The functionality of normal and defective germ cells that determines their probability of fertilization

Using the principles of linear algebra, we can mathematically expect how consecutive fragmentations influence the proportion of the cancer cells in the progeny. Suppose that $f_{3}>\max\left(f_{1}\exp\left[-\mu_{1}T_{F}\right],\;f_{2}\exp\left[-\mu_{2}T_{F}\right]\right)$ holds. In this condition, consecutive fragmentation results in a higher proportion of offspring (Propositions 1, 2 in Appendix S1), and fragmentational purging is not allowed (Corollary 1 in Appendix S1). This implies that the higher propensity of tumor cell transmission via fragmentation ($f_{3}$), higher transition rates ($\mu_{1},\mu_{2}$), and longer time required for the maturity after fragmentation ($T_{F}$) are likely to induce fragmentational accumulation. On the other hand, if $f_{1}\exp\left[-\mu_{1}T_{F}\right]>\max\left(f_{2}\exp\left[-\mu_{2}T_{F}\right],\;f_{3}\right)$, the proportion of cancerous APSCs in NF*n* organisms converges to a certain nonnegative value as fragmentation is repeated (Proposition 1). If $f_{2}\exp\left[-\mu_{2}T_{F}\right]>f_{3}$, the same argument applies to DF*n* organisms (Proposition 2). Proof, conditions, and specific examples (Examples S1, S2) of the mathematical arguments are provided in Appendix S1.

### 2.3 Computational Analysis

To analyze the evolutionary dynamics of the diverse fissiparous or budding organisms, models under different sets of parameters were tested. There are three types of models in this study—FRPG, FRAC, BINF. Fragmentation under FRAC (fragmentational accumulation) models elevates the proportions of cancerous cells of progeny. Therefore, cancers of organisms accumulate through consecutive fragmentations. Fragmentation under FRPG (fragmentational purging) models reduces the proportions of cancerous cells of progeny. While fragmentation of FRPG and FRAC assumes asymmetric fragmentation or budding, BINF (binary fission) models illustrate binary fission inspired by planarian fission assuming (nearly) homogeneously distributed tumor cells. Accordingly, binary fission in this model postulates $f_{1}=f_{2}=f_{3}$ implying that all cells are equitably distributed into two fragments. Unlike asymmetric fragmentation or budding, there is no parent–offspring relationship after binary fission: each half becomes the offspring. Thus, two offspring are produced by binary fission rather than a single nascent offspring from budding or asymmetric fragmentation. Since the fragment from binary fission has the same cell composition as the parent, the mature organism should have a higher proportion of cancer cells than the parent (Corollary 2 in Appendix S1). As such, for binary fission accompanied by equitable cell distribution, consecutive binary fission elevates the proportions of the cancers across generations, similar to FRAC models.

FR or UD were appended to the model names depending on which strategy is fitness‐maximizing, where FR and UD stand for fragmentation and unitary development, respectively. For instance, the model name FRAC‐UD implies that consecutive fragmentation elevates cancer cell composition and unitary development is the fitness‐maximizing strategy. The dynamics of the seven models were examined by changing the parameters including juvenile survival probability ($\Delta_{V}$, $\Delta_{W}$), frequency (τ) and severity (δ) of external damage, the effect of genetic diversity ($b_{0}$) (Table S2). In each model, the inactivation of the fragmentation (ϕ=0) implies the absence of the APSCs (θ=0). In such cases, $\mu_{1}$ and $\mu_{2}$ (Equations (1), (2), (3), (4), (5), (6)) solely represent the transition rates of PGCs.

For each model, five strategies of biased replication fidelity (s is either 0.5, 0.75, 1, 1.25, 1.5) were tested. By changing the values of s in Equations (11) and (12), somatic cells can be intensively protected from the mutation at the cost of elevated mutation of PGCs/APSCs and *vice versa*. APSC proliferability (θ) was varied from 0.1 to 0.7 in steps of 0.1 for each model. Fitness surfaces obtained from different levels of fragmentation prevalence (ϕ) and cancer‐suppression capability (I) were recorded, along with cancer prevalence.

FRPG‐FR model postulates a high degree of fragmentational purging by setting $\left[f_{1},f_{2},f_{3}\right]=\left[5,1,0.5\right]$ and $\left[T_{U},T_{F}\right]=\left[35,15\right]$. NU and ND organisms suffer fitness reduction (e.g., predation, malnutrition) during the juvenile period that NF*i* and DF*i* organisms do not need to sustain ($\left[\Delta_{V},\Delta_{W}\right]=\left[0.7,0.5\right]$). This model would be pertinent to species whose budding is formed at tumor‐free regions with long and susceptible larval periods. Other parameters and details are available in the MATLAB codes.

**FIGURE 5:**
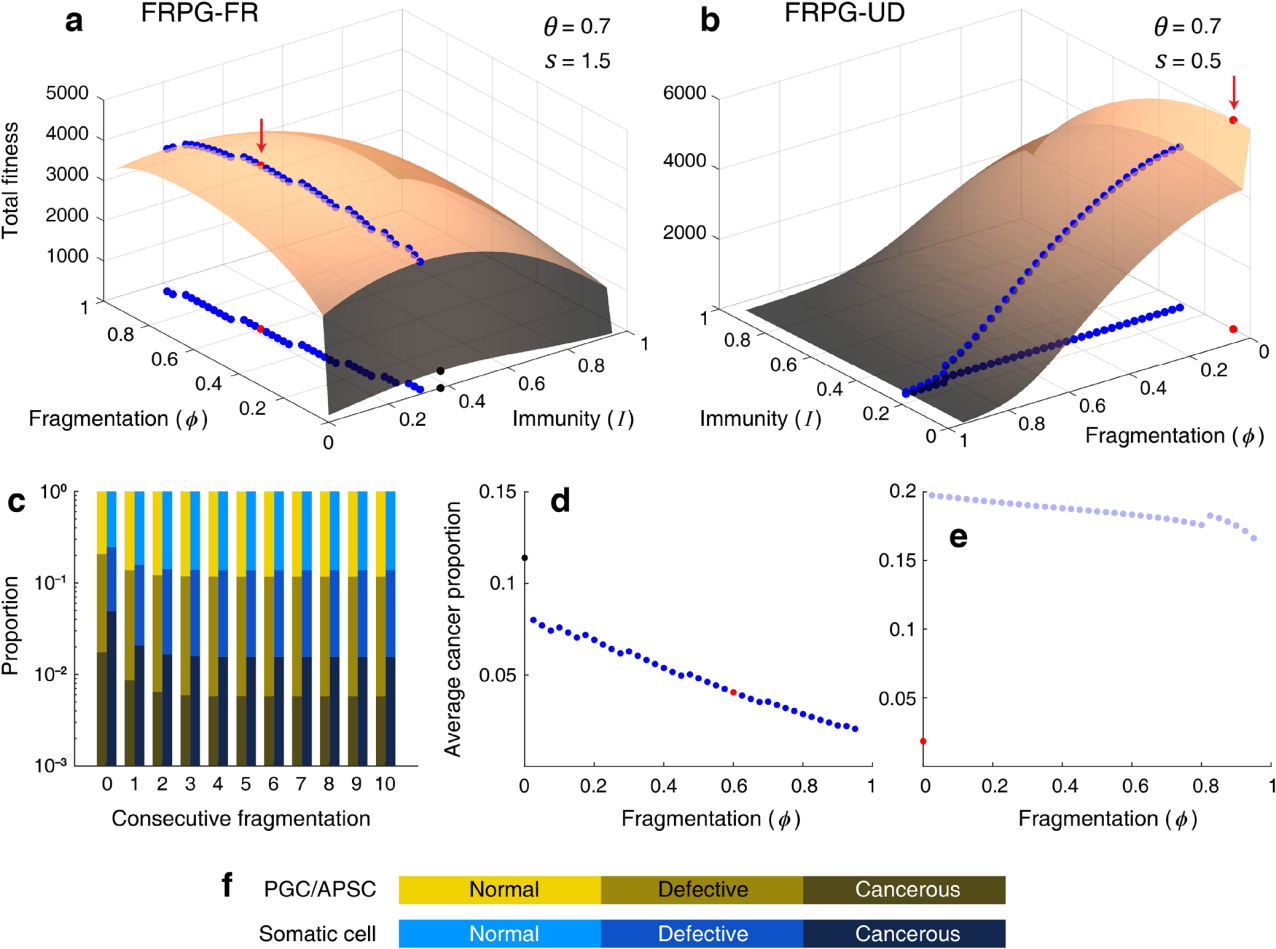
The results of FRPG‐FR and FRPG‐UD model when maximal fitness is achieved. (a) The overall fitness of the populations adopting different reproductive strategies was evaluated across the parameters of the FRPG‐FR model (Video S1). The translucent surface illustrates the overall fitness at the given parameters. Blue dots on the surface mark the maximized fitness for each fragmentation value (ϕ) with the corresponding optimal cancer‐suppression capability (immunity, I). The red dot on the surface represents the globally maximal fitness on the surface. The black dot represents the maximal fitness when fragmentation is inhibited (ϕ=0). The projections of maximal points are also marked on the *xy*‐plane. The globally maximal fitness under θ=0.7 and s=1.5 outcompetes the globally maximal fitness under other conditions. Such a set of parameters postulates the higher APSC proliferation (θ) and APSC replication fidelity (s). Note that optimal immunity in each fragmentation value decreases as fragmentation value increases, as shown by the trend of projected blue markers. (b) The overall fitness of the populations under the FRPG‐UD model was evaluated (Video S2). Note that the configurations of axes are different from (a). Maximal fitness was obtained when the organism did not perform fragmentation with concentrated mutation protection on somatic cells (s=0.5). As it was assumed that APSCs are absent in populations that exclusively perform sexual reproduction, θ does not influence the fitness of populations lacking APSCs. (c) The cell composition of the NU and NF*n* organisms with immunity of optimal fitness under the FRPG‐FR model (the red marker in (a)). Colors represent the proportion of normal, defective, and cancerous cells from the PGC/APSC population (yellow hue) and somatic cells (blue hue). Note that the *y*‐axis is in the log scale. The composition of the NF*n* organism was marked with the number of consecutive fragmentations (*n*), while the NF0 represents the NU organism. Repeated fragmentations reduce the proportions of the cancerous cells across the generations (fragmentational purging). (d) The average cancer prevalence in the population under the conditions that are marked in (a). The blue, red, and black markers in this panel match the conditions of those markers in (a). A higher rate of fragmentation leads to lower average cancer prevalence. (e) Same as (d) for the FRPG‐UD model of (b). The light blue markers imply that fitness on that condition is lower than the fitness obtained from unitary development. (f) Color legend for panel (c).

Throughout diverse values of APSC proliferability in this FRPG‐FR model, the adoption of fragmentation is advantageous for survival (Video S1). The most favorable strategy is to perform fragmentation more frequently than unitary development (ϕ>0.5) with maximized APSC proliferability (θ=0.7) and APSC replication fidelity (s=1.5). As fragmentation became frequent, the corresponding optimal cancer‐suppression level was released (Figure 5a, Video S1). It can be interpreted that prevalent fragmentational purging works as a cancer‐suppression mechanism. Hence, there is less need to sustain the high level of physiologically costly cancer‐suppression level when fragmentation is prevailing.

For the FRPG‐UD model, the organisms are less inflicted by cancer because the exponent of the composition‐to‐fitness function (ε in Equation (15)) was modified from ε=2 of the FRPG‐FR model to ε=1.1. By diminishing the exponent, an increase in cancer cells becomes less detrimental to organismal fitness. Accordingly, the advantage of fragmentational purging becomes less prominent. The external damage is less severe ($\left[\tau ,\delta \right]=\left[0.3,0.1\right]$ in Equation (16)), while the loss of genetic diversity imposes higher fitness reduction in this model ($b_{0}=0.1$ in Equation (22)). Risk during the juvenile period ($\Delta_{V}$, $\Delta_{W}$) and fragmentational values ($f_{1}$, $f_{2}$, $f_{3}$) were modified as well. As a result, though consecutive fragmentation lowers the cancer composition (fragmentational purging), the optimal strategy is to exclusively perform sexual reproduction (Figure 5b). This result indicates that fragmentational purging does not ensure the adoption of fragmentation as an evolutionarily stable strategy. Furthermore, as a general tendency, higher values of fragmentation in FRPG models result in lower cancer prevalence under a wide range of conditions (Figure 5d,e).

**FIGURE 6:**
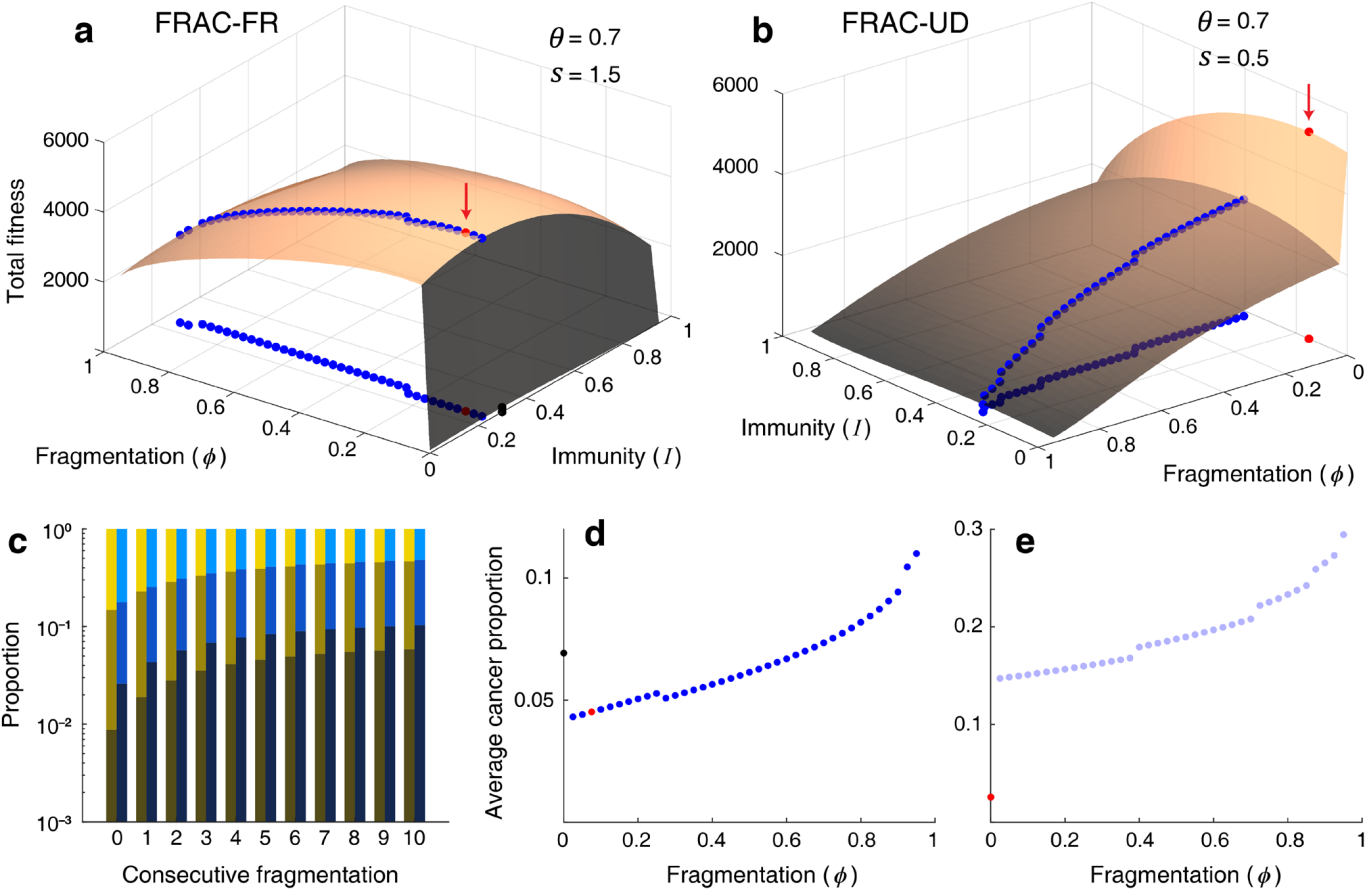
The results of FRAC‐FR and FRAC‐UD model when maximal fitness is achieved. (a) Under the FRAC‐FR model (Video S3), performing fragmentation with maximal APSC proliferability and elevated replication fidelity in APSCs resulted in globally maximal fitness. (b) Under the FRAC‐UD model (Video S4), sexual reproduction without fragmentation resulted in globally maximal fitness. (c) The cell composition of the NU and NF*n* organisms under immunity of optimal fitness from the FRAC‐FR model (the red marker in (a)). Unlike FRPG models, the proportion of cancer rises as consecutive fragmentation is repeated. (d) The average cancer prevalence in the population under the conditions that are marked in (a). Due to the fragmentational accumulation, the higher fragmentation rates induce a higher cancer prevalence. (e) The average cancer prevalence in the population under the conditions that are marked in (b).

On the other hand, consecutive fragmentation elevates cancer composition in the FRAC‐UD model. Under this model, it is optimal to exclude fragmentation with concentrated mutation protection on somatic cells (Figure 6b). By adopting exclusive sexual reproduction, the cancer prevalence can be suppressed to low values compared to those obtained from the corresponding fragmentation‐involved strategies (Figure 6e).

If there is a significant survival risk during the juvenile period and external damage is severe, fragmentation can be selected for though consecutive fragmentation raises the cancer composition, as evidenced by results of the FRAC‐FR (Figure 6a). Unlike FRPG models, higher fragmentation in FRAC models leads to higher cancer prevalence at the optimized cancer‐suppression level (Figure 6d,e).

Different conditions were implemented in BINF‐FRa and BINF‐FRb. BINF‐FRa is characterized by the prevalent external damage and the low survival rate of juvenile organisms established from unitary development. Additionally, it was assumed that loss of genetic diversity stemming from asexual reproduction imposes mild fitness reduction ($b_{0}=0.7$). As a result, fragmentation is favored in this model (Video S5).

Due to the modified fitness function (changes in $\alpha_{N}$, $\alpha_{D}$, $\alpha_{C}$, ε of Equation (15), Table S2), the cancer does not dramatically reduce organismal fitness under the BINF‐FRb model. In this model, fragmentation is favored with concentrated replication fidelity on APSCs (Video S6). Results of BINF‐FRa and BINF‐FRb illustrate two possible evolutionary pressures for the adoption of binary fission.

Reduced risk during juvenility, prominent vulnerability resulting from the loss of genetic diversity, and infrequent external damage lead to dominance of unitary development excluding fragmentation, as supported by BINF‐UD (Video S7). All model results are summarized in Videos [Link], [Link], [Link], [Link], [Link], [Link], [Link], and specific model parameters are presented in Table S2.

## 3 Discussion

Although fragmentation (including budding) is not a common mode of reproduction in animals, a comparative analysis of cancer susceptibility and cancer suppression therein would expand our understanding of cancer from an evolutionary perspective. Considering the existence of the APSCs, verified or presumptive, in fragmentational species, the APSCs could be the prerequisite for performing fissiparity or budding. The APSCs are associated with high regenerative capability, while sharing multiple essential traits with cancer stem cells.

Two counterbalancing effects of carcinogenesis are involved in the reproduction by fragmentation. Although fragmentation explicitly delivers the parental somatic cancer cells to progeny, it can reduce the number of cell replications (and accordingly time) required to reach maturity. To simplify the situation, suppose that all cells are viable until maturity. Given that the cell number at maturity is $N_{M}$, a zygote and its progeny cells need to replicate $\log_{2}N_{M}$ times to reach maturity. Given that the offspring contains $rN_{M}$ cells after fragmentation (0<r≤0.5), the required number of the replication is $\log_{2}\left(rN_{M}\right)$. For example, if a mature organism is composed of 1024 (= 2^10^) cells, then a zygote needs to replicate 10 times to reach that size. On the other hand, if a detached fragment contains 128 (= 2^7^ where r=1/8) cells, each cell needs to divide 3 times. The reduction in replication number culminates in binary fission (r=0.5). Under this simplified framework, only a single replication of each cell suffices to establish an integral organism after binary fission. Even though binary fission assuming equitable cell distribution will intensify the cancer risk of offspring (Corollary 2), the time required for maturation after fission will be substantially reduced than the maturation from a zygote (Ivankovic et al. 2019; Stocchino and Manconi 2013). By all means, this postulation does not consider the lifespan of cells and the precise developmental process toward maturity. Nonetheless, it is indisputable that offspring produced via fragmentation benefit from fewer cell replications or shorter maturation periods (implemented in the models by $T_{U}$, $T_{F}$, $\Delta_{V}$, $\Delta_{W}$). As the number of stem cell replications is an evident risk factor for cancer (Tomasetti and Vogelstein 2015), fragmentation may reduce the offspring's cancer risk.

How cancerous or defective cells are transmitted to progeny combined with the reduced number of cell replications determines whether fragmentation elevates the cancer prevalence of the progeny. Depending on such conditions, consecutive fragmentation may suppress or raise the cancer prevalence (fragmentational purging or accumulation) as investigated in Propositions 1, 2, and Corollary 1. Mathematical analyses imply that a lower rate of vertical cancer transmission ($f_{3}$) and the reduced number of cell replications after fragmentation ($T_{F}$) are likely to induce fragmentational purging. Frequent fragmentational purging functions to suppress cancer so that the burden of physiological cancer suppression is relieved (Figure 5a, Video S1). However, fragmentational purging does not ensure the adoption of fragmentation (FRPG‐UD), nor does fragmentational accumulation necessarily prevent fragmentation (FPAC‐FR). Despite the fragmentational accumulation, a low survival rate during juvenility ($\Delta_{V}$, $\Delta_{W}$) facilitates the adoption of fragmentation as shown in FRAC‐FR and BINF‐FRa models. In contrast, the benignity of the tumor was postulated in the FRPG‐UD model, implying that fragmentational purging may not be a merit if the tumor is not a serious threat.

Another consequence of the fragmentation is the loss of genetic diversity. Sexual reproduction allows the production of offspring with diversified traits, endowing adaptive advantage under changing environments (Becks and Agrawal 2012; Bürger 1999). Deleterious mutations can be eliminated during the course of sexual reproduction (Muller 1964). Particularly, sexual reproduction could confer protection from horizontally transmissible cancers (Thomas et al. 2019). This factor was implemented in the model by changing the coefficient $b_{0}$ in Equation (22).

A certain *Hydra* species, *Hydra oligactis* and *P. robusta* , spontaneously develop tumor (Boutry et al. 2023), but not all progeny budded from tumoral parents have transmitted tumor (Boutry et al. 2022). Even in the presence of tumors, they still can produce their progeny sexually or asexually (Tissot et al. 2024; Domazet‐Lošo et al. 2014; Boutry et al. 2022). Consecutive vertical cancer transmission of *H. oligactis* appears to elevate the cancer proportion of the offspring (Tissot et al. 2024), comparable to fragmentational accumulation. Furthermore, the bearing of tumors may alter the reproductive strategy of the host *Hydra* such as reproductive age or budding rates (Tissot et al. 2024; Boutry et al. 2022). Similarly, tumoral planaria still can reproduce asexually (Lange 1966).

Consecutive fragmentation mediated by binary fission postulating equal distribution of cells ($f_{1}=f_{2}=f_{3}$) is expected to elevate cancer composition throughout generations (Corollary 2). Despite this drawback, results from BINF‐FRa and BINF‐FRb illustrate possible evolutionary pressures for adopting binary fission. In both models, maximal fitness is achieved when APSCs are strongly protected from mutation at the cost of somatic cell mutation risk. In accordance with this finding, stem cells of *Schmidtea mediterranea* are selectively protected from carcinogenesis (Van Roten et al. 2018; Mihaylova et al. 2018). However, within the same species of *S. mediterranea* , different strains exhibit different modes of sexual and asexual reproduction strategies (Zayas et al. 2005). Moreover, exposure to cadmium has different effects on planarian species. For example, *S. mediterranea* is resistant to cadmium‐induced carcinogenesis, while *Dugesia dorotocephala* and *Dugesia tigrina* are vulnerable (Van Roten et al. 2018). While it was assumed in the model that cells are equitably distributed to the two fragments via binary fission, either of the fragments could be purged as the planarian tumors of *Dugesia etrusca* and *Dugesia ilvana* are usually located at the posterior tip of the body (Lange 1966). As a result of the transverse fission, the progeny developed from the anterior fragment was not afflicted by the parental tumor (Lange 1966), reminiscent of fragmentational purging. In this case, the FRPG‐FR model assuming $f_{1}>f_{3}$ for the anterior fragment would be suitable to describe the evolutionary dynamics of those planarians.

Though comprehensive data is not available, I examined the cancer prevalence and regeneration capability of diverse animal (Metazoa) taxa which perform fragmentation after maturation. Intriguingly, while cancer is a prevailing disease among the wide range of animal taxa (Aktipis et al. 2015), there is no evidence of spontaneous cancer under natural conditions in some fissiparous or budding animals including starfish, sea anemone, and acorn worms (Table S1). It is also noteworthy that sponges and placozoans do not develop tumors even after exposure to high radiation (Fortunato et al. 2021, 2025). On the other hand, planarians, *Hydra*, corals, and brittle stars are susceptible to cancer though they perform fragmentation (Aktipis et al. 2015).

The proposed model can provide a possible explanation for different degrees of cancer susceptibility in fragmentational animals depending on cell replication number and degree of vertical cancer transmission rates which are quantitatively measurable (Tissot et al. 2024; Domazet‐Lošo et al. 2014). Cancer‐suppression capability (I in Equations (11), (12)) can be investigated as well. For example, many animals in which no sign of cancer was reported such as starfish (Kicha et al. 2010), sponges (Calcabrini et al. 2017), annelids (Macedo et al. 2021), sea anemones (Soletti et al. 2008) produce diverse anticancer substances.

Obviously, the absence of reports on cancer could be the consequence of insufficient observation: subsequent investigation may identify the naturally occurring tumors in those animals that are reportedly cancer‐resistant. Interspecific variation complicates the generalization of the relationship between fragmentation and cancer susceptibility. Further investigation is required to clarify how modes of reproduction influence the evolution of cancer (Table 2).

**TABLE 2:** Future research questions.

Do animals that can perform fragmentation or budding have adult primordial stem cells (APSCs)?
Are fissiparous animals generally robust against cancer?
What is the required number of cell replications after fragmentation compared to the unitary development?
How much of the parental cancer cells are included in the fragment for asexual reproduction?
Are APSCs heavily protected from mutation (compared to somatic bystander cells)?
Does mutation in APSCs lead to carcinogenesis in fragmentational animals?
Does possession of APSCs inhibit a higher degree of differentiation across animal taxa?

The high degree of differentiation ensuring diverse cell types may not reliably coincide with the multipotency of the adult cells including the capability to dedifferentiate (epimorphosis) or transdifferentiate (morphallaxis) (Cho et al. 2024). Corroborating to this speculation, the organisms that can perform fragmentation up to date exhibit relatively simple body plans composed of a minor number of cell types (Table S1). Namely, possession of APSCs at the cost of sophisticated cell differentiation could be a suitable strategy for certain animals.

Extensive differentiation requires elaborate developmental mechanisms, the mutation at which would impose oncogenic transformation (cell specialization trade‐offs *sensu* Hammarlund et al. (2020)). As the number of the molecular pathways of the cells that control the proliferation and development increases, the risk of oncogenic mutations at either of those pathways would accordingly increase (Jacques et al. 2022). If all highly differentiated somatic cells are endowed with multipotency including transdifferentiation or dedifferentiation, one may speculate that the risk of cancer will dramatically escalate (Cho et al. 2024; Jacques et al. 2022). This would be the reason that animals with complex body plans are incapable of performing fragmentation and their regenerative capability is limited. *Polypodium hydriforme* could be an example of such a trade‐off between differentiation and regeneration (Table S1). Compared to other cnidarians, *P. hydriforme* has highly differentiated cell types (Raikova 2008). Though *P. hydriforme* can reproduce by fission, this animal does not exhibit remarkable regeneration (Raikova 2008).

To my knowledge, this is the first analytical model that investigates the link between asexual reproduction via fragmentation and evolutionary dynamics of cancer susceptibility by incorporating the number of cell replications, influence of APSCs, and vertical transmission extent of cancer cells. This study demonstrates various evolutionary pathways that fragmentation can be selected for and the corresponding cancer prevalence. Further examination of fissiparous or budding animals would verify the validity of the proposed model (Table 2) and provide insights for medical applications.

Germ cells, stem cells, and cancer stem cells have multiple features in common, particularly under the perspective of germline multipotent programs. Regeneration, cell type diversification, and oncogenicity would be accordingly intertwined (Jacques et al. 2022). Mammals would have favored complex body plans including neural systems by releasing regeneration, while *Hydra* would have pursued regeneration and budding at the cost of highly specialized cells. This perspective enables us to appreciate cancer as the outcome of the evolutionary trade‐offs (Jacqueline et al. 2017).

## Conflicts of Interest

The author declares no conflicts of interest.

## Supporting information


Appendix S1.

**Video S1.** Results of FRPG‐FR model.

**Video S2.** Results of FRPG‐UD model.

**Video S3.** Results of FRAC‐FR model.

**Video S4.** Results of FRAC‐UD model.

**Video S5.** Results of BINF‐FRa model.

**Video S6.** Results of BINF‐FRb model.

**Video S7.** Results of BINF‐UD model.

## Data Availability

The code for performing the computational analysis in this study is provided in Zenodo: https://zenodo.org/records/12789477.
