# Supplementary material for: Vertical Cancer Transmission via Asexual Fragmentation and Associated Cancer Prevalence
Source: Evol Appl. 2025 May 21;18(5):e70111. doi: 10.1111/eva.70111 (PMC12093053; doi:10.1111/eva.70111)
Supplement: Supplementary file 1 — Appendix S1. [file EVA-18-e70111-s001.pdf]

Supporting Information

Vertical Cancer Transmission via Asexual Fragmentation and Associated Cancer Prevalence

Jibeom Choi[1,2,*]

[1]Department of Applied Mathematics, Kyung Hee University, Yongin, Republic of Korea

[2]School of Computational Sciences, Korea Institute for Advanced Study, Seoul, Republic of Korea

*Corresponding author: snu10@snu.ac.kr

Additional Model Explanation

Modes of reproduction and population dynamics

Suppose that an NU organism reproduces sexually. Π^V, Ψ^V, Γ^V are the proportions of the normal, defective, cancerous PGCs/APSCs of mature NU organisms. It was assumed that the composition of germ cells (gametes) is identical to that of PGCs/APSCs. r_1, r_2, r_3 are the coefficients that determine the differential fertilization success of normal, defective, cancerous germ cells (Equations [17]–[21], [23]). As such, it was postulated that $r_1 \geq r_2 \geq r_3$. Given that sexual reproduction has occurred, let U_1^V denote the probability that an offspring of an NU organism is established from a normal zygote. The following equation was used to deduce such probability.

$$U_1^V = \frac{r_1 \Pi^V}{r_1 \Pi^V + r_2 \Psi^V + r_3 \Gamma^V}. \qquad [S1]$$

Similarly, let U_2^V denote the probability that the offspring of an NU organism is established from a defective zygote after sexual reproduction.

$$U_2^V = \frac{r_2 \Psi^V}{r_1 \Pi^V + r_2 \Psi^V + r_3 \Gamma^V}. \qquad [S2]$$

NFi organisms can reproduce sexually as well. $U_{1,i}^Z$ and $U_{2,i}^Z$ are the probability that a zygote generated by the sexual reproduction of NFi organisms is normal and defective, respectively.

$$U_{1,i}^Z = \frac{r_1 \Pi_i^Z}{r_1 \Pi_i^Z + r_2 \Psi_i^Z + r_3 \Gamma_i^Z}, \qquad [S3]$$

$$U_{2,i}^Z = \frac{r_2 \Psi_i^Z}{r_1 \Pi_i^Z + r_2 \Psi_i^Z + r_3 \Gamma_i^Z} \qquad [S4]$$

where Π_i^Z, Ψ_i^Z, Γ_i^Z are the proportions of the normal, defective, cancerous germ cells of NFi organisms.

Fertilization of the cancerous germ cell leads to the embryonic lethality in all cases.

Similar equations can be used to estimate the probability that DU and DFi organisms produce normal or defective zygotes via sexual reproduction. As there is no normal cell in DU and DFi organisms, all zygotes from those organisms are defective.

For Animations S1–S7, the panels of the respective rows represent the conditions of s from 0.5 (the first row) to 1.5 (the last row).

The mutation degree and immunity (cancer-suppression capability)

The equation of the baseline transition rate (Equations [11], [12]) was formulated as follows.

$$\eta(I) = b + (a - b)(1 - I). \quad \text{[S5]}$$

Here, $a = 0.01$ and $b = 0.0001$. Hence, for a range of I from 0 to 1, $\eta(I)$ is from a to b. In other words, $\eta(I)$ reaches the maximal value of a at $I = 0$, and the minimal value of b at $I = 1$.

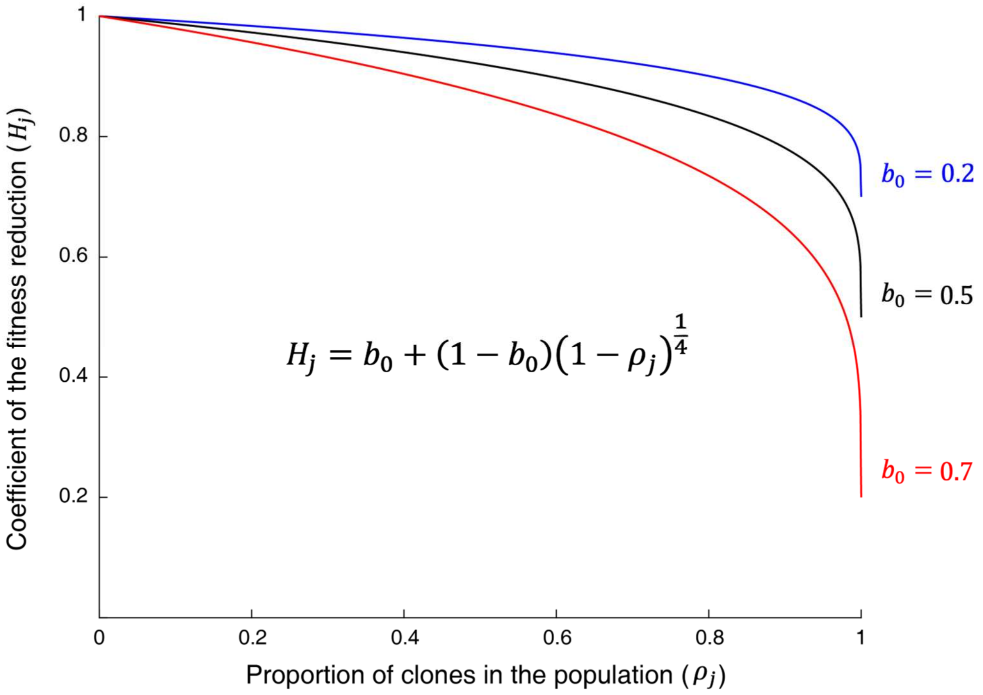

Figure S1. The graph of Equation [22].

Population variation

Considering the definition of reproductive success which could be the proxy of fitness, the sum of the offspring produced by the parental organisms multiplied by the parental fitness should be the population size of the subsequent generation. Let this size be referred to as the untransformed population size. In the natural system, however, the total population size in each generation (P_j's) will change in a gradual manner. To this end, it was posited that

$$P_{j+1} = \Theta\left(\frac{P_{j+1}^*}{P_j}\right)P_j \quad \text{[S6]}$$

where P_{j+1}^* is the untransformed population size of (j + 1)-th generation. In addition,

$$\Theta(r) = \frac{2d}{\exp[-k(r-1)] + 1} + 1 - d. \quad \text{[S7]}$$

In this study, $d = 0.08$ and $k = 2$. Under these parameters, $0.9391 \leq \Theta(r) \leq 1.08$ for $r \geq 0$. Trivially, $\Theta(1) = 1$. This transformation prevents abrupt changes in population sizes (Figure S2).

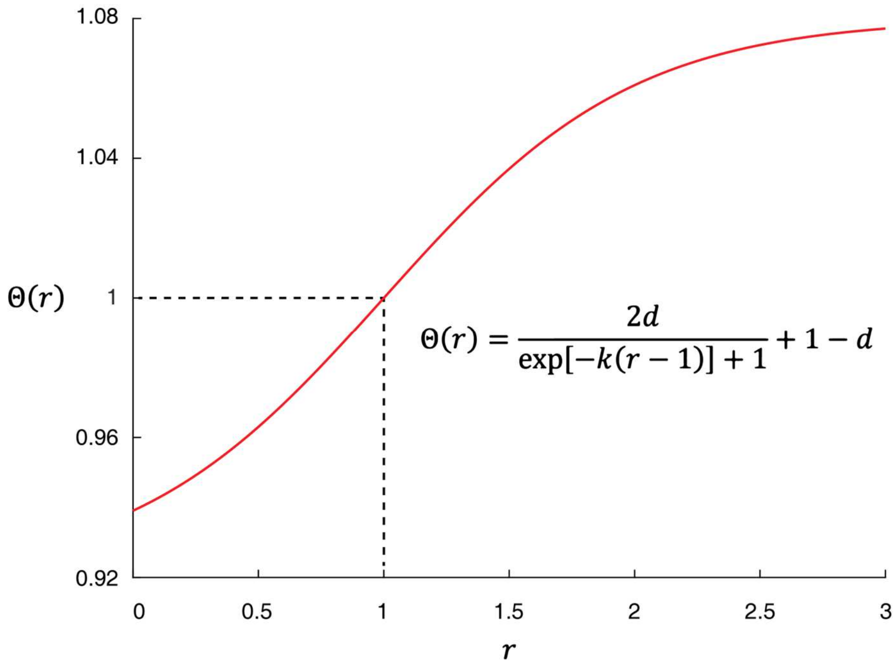

Figure S2. The graph of Equation [S7].

Mathematical Analysis

Proposition 1

Suppose that $f_1 \exp[-\mu_1 T_F]$, $f_2 \exp[-\mu_2 T_F]$, f_3 are pairwise distinct. If $f_1 \exp[-\mu_1 T_F] > \max(f_3, f_2 \exp[-\mu_2 T_F])$, then the proportion of PGCs/APSCs will converge to a certain equilibrium whose elements are nonzero as consecutive fragmentation of NFn organisms tends to infinity. If $f_3 > \max(f_1 \exp[-\mu_1 T_F], f_2 \exp[-\mu_2 T_F])$, then the proportions of cancer cells in PGCs/APSCs will tend to 1 as consecutive fragmentation of NFn organisms tends to infinity. This proposition requires the constraint that σ is positive (refer to Equation [S19]).

Proof

Based on general solutions of Equations [1]–[3], define the mutation matrix $\mathbf{\Omega}$ as follows.

$$\mathbf{\Omega} = \begin{bmatrix} \exp[-\mu_1 T_F] & 0 & 0 \\ \frac{\mu_1}{\mu_2 - \mu_1}(\exp[-\mu_1 T_F] - \exp[-\mu_2 T_F]) & \exp[-\mu_2 T_F] & 0 \\ 1 - \frac{1}{\mu_2 - \mu_1}(\mu_2 \exp[-\mu_1 T_F] - \mu_1 \exp[-\mu_2 T_F]) & 1 - \exp[-\mu_2 T_F] & 1 \end{bmatrix}. \quad [S8]$$

Let Π_i^M, Ψ_i^M, Γ_i^M represent the proportion of normal, defective, cancerous PGCs/APSCs, respectively, of mature NFi or DFi organisms. The PGC/APSC composition of an organism established from fragmentation is

$$\begin{bmatrix} \Pi_{i+1}^M \\ \Psi_{i+1}^M \\ \Gamma_{i+1}^M \end{bmatrix} = \alpha_i \mathbf{\Omega} \mathbf{F} \begin{bmatrix} \Pi_i^M \\ \Psi_i^M \\ \Gamma_i^M \end{bmatrix} \quad [S9]$$

where α_i is the scaling factor that satisfies the condition of $\Pi_{i+1}^M + \Psi_{i+1}^M + \Gamma_{i+1}^M = 1$ and

$$\mathbf{F} = \begin{bmatrix} f_1 & 0 & 0 \\ 0 & f_2 & 0 \\ 0 & 0 & f_3 \end{bmatrix}. \quad [S10]$$

The eigenvalues of $\mathbf{\Omega F}$ are

$$\lambda_1 = f_1 \exp[-\mu_1 T_F], \quad [S11]$$

$$\lambda_2 = f_2 \exp[-\mu_2 T_F], \quad [S12]$$

$$\lambda_3 = f_3. \quad [S13]$$

For simplicity, take

$$m_1 = \exp[-\mu_1 T_F]\,, \qquad \text{[S14]}$$

$$m_2 = \exp[-\mu_2 T_F]\,. \qquad \text{[S15]}$$

The corresponding eigenvectors are

$$\mathbf{v}_1 = \begin{bmatrix} (f_1 m_1 - f_3)(f_1 m_1 - f_2 m_2)(\mu_2 - \mu_1)/\sigma \\ (f_1 m_1 - f_3)(m_1 - m_2) f_2 \mu_1/\sigma \\ f_3 \end{bmatrix}, \qquad \text{[S16]}$$

$$\mathbf{v}_2 = \begin{bmatrix} 0 \\ f_2 m_2 - f_3 \\ f_3(1 - m_2) \end{bmatrix}, \qquad \text{[S17]}$$

$$\mathbf{v}_3 = \begin{bmatrix} 0 \\ 0 \\ 1 \end{bmatrix} \qquad \text{[S18]}$$

where

$$\sigma = f_1 m_1 (\mu_1 - \mu_2 + m_1 \mu_2 - m_2 \mu_1) - f_2\big(m_1 \mu_1 - m_2 \mu_2 - m_1 m_2\,(\mu_1 - \mu_2)\big). \qquad \text{[S19]}$$

Utilizing the eigendecomposition,

$$\mathbf{\Omega F} = \mathbf{PDP}^{-1} \qquad \text{[S20]}$$

where **P** is a 3×3 matrix whose columns are eigenvectors and **D** is a diagonal matrix containing eigenvalues.

$$\mathbf{P} = [\mathbf{v}_1\ \mathbf{v}_2\ \mathbf{v}_3], \qquad \text{[S21]}$$

$$\mathbf{D} = \begin{bmatrix} \lambda_1 & 0 & 0 \\ 0 & \lambda_2 & 0 \\ 0 & 0 & \lambda_3 \end{bmatrix}. \qquad \text{[S22]}$$

Therefore,

$$\begin{bmatrix} \Pi_{i+1}^M \\ \Psi_{i+1}^M \\ \Gamma_{i+1}^M \end{bmatrix} = \alpha_i \mathbf{\Omega F} \begin{bmatrix} \Pi_i^M \\ \Psi_i^M \\ \Gamma_i^M \end{bmatrix} = \alpha_i \mathbf{PDP}^{-1} \begin{bmatrix} \Pi_i^M \\ \Psi_i^M \\ \Gamma_i^M \end{bmatrix} \qquad \text{[S23]}$$

and

$$\begin{bmatrix}\Pi_n^M\\\Psi_n^M\\\Gamma_n^M\end{bmatrix}=\left(\prod_{i=0}^{n-1}\alpha_i\right)\mathbf{P}\mathbf{D}^n\mathbf{P}^{-1}\begin{bmatrix}\Pi_0^M\\\Psi_0^M\\\Gamma_0^M\end{bmatrix}=\left(\prod_{i=0}^{n-1}\alpha_i\right)(c_1\lambda_1^n\mathbf{v}_1+c_2\lambda_2^n\mathbf{v}_2+c_3\lambda_3^n\mathbf{v}_3) \quad [S24]$$

where coefficients c_1, c_2, c_3 are determined by the initial condition.

For T_U, take

$$m_1^* = \exp[-\mu_1 T_U], \quad [S25]$$

$$m_2^* = \exp[-\mu_2 T_U], \quad [S26]$$

$$\boldsymbol{\Omega}^* = \begin{bmatrix}\exp[-\mu_1 T_U] & 0 & 0\\ \frac{\mu_1}{\mu_2-\mu_1}(\exp[-\mu_1 T_U]-\exp[-\mu_2 T_U]) & \exp[-\mu_2 T_U] & 0\\ 1-\frac{1}{\mu_2-\mu_1}(\mu_2\exp[-\mu_1 T_U]-\mu_1\exp[-\mu_2 T_U]) & 1-\exp[-\mu_2 T_U] & 1\end{bmatrix}. \quad [S27]$$

Then,

$$\begin{bmatrix}\Pi_0^M\\\Psi_0^M\\\Gamma_0^M\end{bmatrix}=\begin{bmatrix}\Pi^V\\\Psi^V\\\Gamma^V\end{bmatrix}=\boldsymbol{\Omega}^*\begin{bmatrix}1\\0\\0\end{bmatrix} \quad [S28]$$

for NFi organisms, and

$$\begin{bmatrix}\Pi_0^M\\\Psi_0^M\\\Gamma_0^M\end{bmatrix}=\begin{bmatrix}\Pi^W\\\Psi^W\\\Gamma^W\end{bmatrix}=\boldsymbol{\Omega}^*\begin{bmatrix}0\\1\\0\end{bmatrix} \quad [S29]$$

for DFi organisms. Note that Π_0^M, Ψ_0^M, Γ_0^M are nonnegative values for NU organisms while $\Pi_0^M = 0$ for DU organisms.

Consequently, the convergence of the composition as the generation tends to infinity is determined by the size of the eigenvalues. If $\lambda_1 = f_1 m_1$ is the principal eigenvalue ($|\lambda_1| > \max(|\lambda_2|, |\lambda_3|)$), then the composition of NFi organisms will converge to

$$\lim_{i\to\infty}\begin{bmatrix}\Pi_i^M\\\Psi_i^M\\\Gamma_i^M\end{bmatrix}=h_N\begin{bmatrix}(f_1m_1-f_3)(f_1m_1-f_2m_2)(\mu_2-\mu_1)/\sigma\\(f_1m_1-f_3)(m_1-m_2)f_2\mu_1/\sigma\\f_3\end{bmatrix} \quad [S30]$$

where h_N is a scaling factor so that $\lim_{i\to\infty}(\Pi_i^M+\Psi_i^M+\Gamma_i^M)=1$. Due to the initial condition of the NFi organisms, $c_1 \neq 0$ if $f_1 m_1$ is the principal eigenvalue.

Otherwise, if $\lambda_3 = f_3$ is the principal eigenvalue,

$$\lim_{i\to\infty}\begin{bmatrix}\Pi_i^M\\ \Psi_i^M\\ \Gamma_i^M\end{bmatrix} = \begin{bmatrix}0\\0\\1\end{bmatrix} \qquad [S31]$$

indicating that all PGCs/APSCs of NF*i* organisms will become cancerous as consecutive fragmentations proceed across generations. In this condition, assuming $c_3 = 0$ induces contradiction, which ensures that $c_3 \neq 0$ by the reduction to absurdity.

□

Proposition 2

Suppose that $f_1 \exp[-\mu_1 T_F]$, $f_2 \exp[-\mu_2 T_F]$, f_3 are pairwise distinct. If $f_2 \exp[-\mu_2 T_F] > f_3$, then the proportion of PGCs/APSCs will converge to a certain equilibrium whose proportions of normal and defective PGCs/APSCs are nonzero as consecutive fragmentation of DF*n* organisms tends to infinity. If $f_3 > f_2 \exp[-\mu_2 T_F]$, then the proportions of cancer cells in PGCs/APSCs will tend to 1 as consecutive fragmentation of DF*n* organisms tends to infinity.

Proof

As in the proof of Proposition 1, the general solution of Equation [S24] holds. However, as $\Pi_0^M = 0$ for DU organisms, $c_1 = 0$ in Equation [S24]. Moreover, as $\Psi_0^M \neq 0$, it follows that $c_2 \neq 0$. Hence, $f_2 \exp[-\mu_2 T_F] > f_3$ implies that the proportions of the DF*n* organisms converges to

$$\lim_{i\to\infty}\begin{bmatrix}\Pi_i^M\\ \Psi_i^M\\ \Gamma_i^M\end{bmatrix} = h_D\begin{bmatrix}0\\ f_2 m_2 - f_3\\ f_3(1-m_2)\end{bmatrix} \qquad [S32]$$

where h_D is a scaling factor so that $\lim_{i\to\infty}(\Pi_i^M + \Psi_i^M + \Gamma_i^M) = 1$.

If $f_2 \exp[-\mu_2 T_F] < f_3$,

$$\lim_{i\to\infty}\begin{bmatrix}\Pi_i^M\\ \Psi_i^M\\ \Gamma_i^M\end{bmatrix} = \begin{bmatrix}0\\0\\1\end{bmatrix} \qquad [S33]$$

holds for DF*n* organisms. In this condition, assuming $c_3 = 0$ results in contradiction, which guarantees that $c_3 \neq 0$ by the reduction to absurdity.

□

Definition 1

Fragmentational purging is defined as

$$\Gamma_{i+1}^{M} < \Gamma_{i}^{M} \qquad [S34]$$

for all $i \geq 0$ where $\Gamma_0^M = \Gamma^V$ or $\Gamma_0^M = \Gamma^W$.

Likewise, fragmentational accumulation is defined as

$$\Gamma_{i+1}^{M} > \Gamma_{i}^{M} \qquad [S35]$$

for all $i \geq 0$ where $\Gamma_0^M = \Gamma^V$ or $\Gamma_0^M = \Gamma^W$.

Corollary 1

If $f_1 \exp[-\mu_1 T_F] > \max(f_3,\ f_2 \exp[-\mu_2 T_F])$ holds where Proposition 1 is applicable, fragmentational purging of NFn organisms is not allowed when

$$\lim_{i\to\infty} \Gamma_i^M = h_N f_3 > \Gamma^V. \qquad [S36]$$

where h_N is the scaling factor of Equation [S30].

Equivalently, the prerequisite (not indicating the sufficient condition) for fragmentational purging of NFn organisms under this condition is

$$\lim_{i\to\infty} \Gamma_i^M = h_N f_3 < \Gamma^V. \qquad [S37]$$

For DFn organisms satisfying $f_2 \exp[-\mu_2 T_F] > f_3$ so that Proposition 2 is applicable, fragmentational purging of DFn organisms is not allowed when

$$\lim_{i\to\infty} \Gamma_i^M = h_D f_3 (1 - m_2) > \Gamma^V. \qquad [S38]$$

where h_D is the scaling factor of Equation [S32].

Equivalently, the prerequisite for fragmentational purging of DFn organisms under this condition is

$$\lim_{i\to\infty} \Gamma_i^M = h_D f_3 (1 - m_2) < \Gamma^V. \qquad [S39]$$

If $f_3 > \max(f_1 \exp[-\mu_1 T_F],\ f_2 \exp[-\mu_2 T_F])$ holds where Propositions 1 and 2 are applicable, fragmentational purging is not allowed for NFn and DFn organisms.

□

Corollary 2

Binary fission postulating that $f_1 = f_2 = f_3$ results in fragmentational accumulation, and consecutive fragmentation leads to saturation of cancerous PGCs/APSCs.

Proof

If we postulate $f_1 = f_2 = f_3 = 1$, the following equation holds.

$$\begin{bmatrix} \Pi_{i+1}^M \\ \Psi_{i+1}^M \\ \Gamma_{i+1}^M \end{bmatrix} = \mathbf{\Omega} \begin{bmatrix} \Pi_i^M \\ \Psi_i^M \\ \Gamma_i^M \end{bmatrix}. \qquad [S40]$$

which implies that $\alpha_i = 1$. Moreover, $f_3 > \max(f_1 \exp[-\mu_1 T_F],\ f_2 \exp[-\mu_2 T_F])$ holds.

Solving this equation shows that

$$\Gamma_{i+1}^M = \left(1 - \frac{\mu_2 m_1 - \mu_1 m_2}{\mu_2 - \mu_1}\right) \Pi_i^M + (1 - m_2)\Psi_i^M + \Gamma_i^M. \qquad [S41]$$

Hence, $\Gamma_{i+1}^M > \Gamma_i^M$.

□

Example S1

Suppose that $[\mu_1,\ \mu_2] = [0.1,\ 0.15]$, $[T_U,\ T_F] = [20, 10]$, $[f_1,\ f_2,\ f_3] = [5, 3, 0.5]$. As such, $f_1 \exp[-\mu_1 T_F] > \max(f_3,\ f_2 \exp[-\mu_2 T_F])$ and $f_2 \exp[-\mu_2 T_F] > f_3$ hold. It can be expected from Propositions 1 and 2 that proportions of normal, defective, and cancerous APSCs of mature progeny converge toward a certain equilibrium. As shown in Figure S3, the actual proportions of those cells estimated from Equation [S9] are compliant with Propositions 1 and 2. In this case, fragmentational purging is observed in both NFn and DFn organisms.

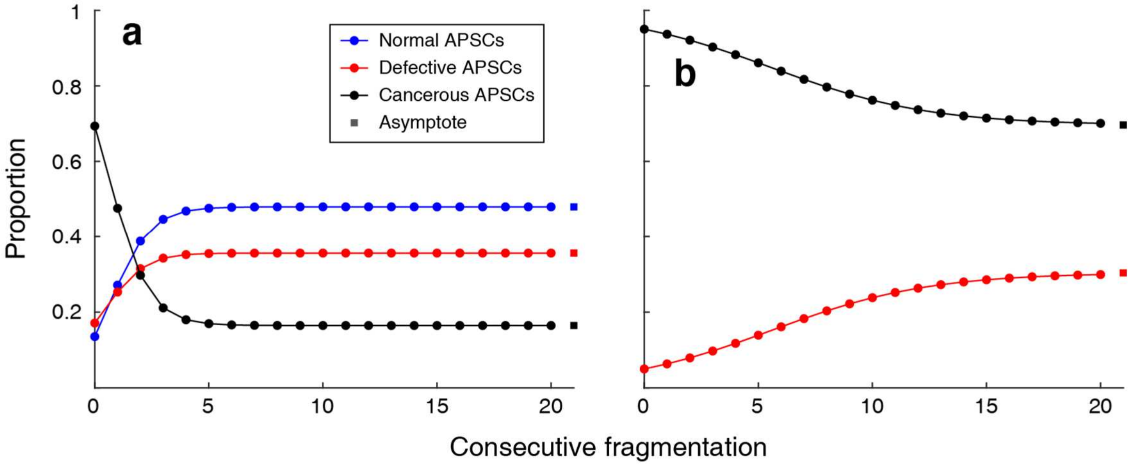

Figure S3. The proportions of APSCs as consecutive fragmentation proceeds in Example S1.

(a) The APSC proportions of NFn organisms. The proportion at the zeroth fragmentation refers to the values of NU organisms. The square markers of the corresponding colors indicate the theoretical asymptotes obtained from Propositions 1 and 2. As the proportion of cancer cells decreases as fragmentation is repeated, this example describes fragmentational purging.

(b) The APSC proportions of DFn organisms. The dynamics reveal the fragmentational purging.

Example S2

In this example, all conditions are identical to those of Example S1 except that $[f_1,\ f_2,\ f_3] = [5, 3, 1]$. Hence, $f_1 \exp[-\mu_1 T_F] > \max(f_3,\ f_2 \exp[-\mu_2 T_F])$, while $f_3 > f_2 \exp[-\mu_2 T_F]$ holds. As expected from Proposition 1, the proportions of normal, defective, and cancerous APSCs of mature NFn progeny converge toward a certain equilibrium composed of nonnegative values (Figure S4a). On the other hand, all APSCs of mature DFn progeny become cancerous as consecutive fragmentation continues (Figure S4b).

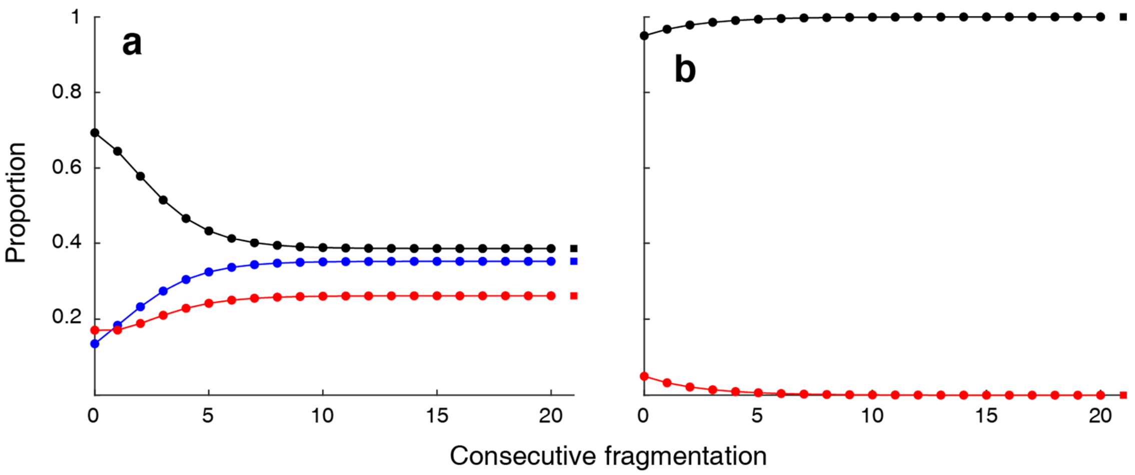

Figure S4. The proportions of APSCs as consecutive fragmentation proceeds in Example S2.
(a) The APSC proportions of NFn organisms. The color scheme is identical to Figure S3.
(b) The APSC proportions of DFn organisms. The dynamics reveal the fragmentational accumulation.

Table S1. Cancer susceptibility and regenerative capability of animal taxa that reproduce via fragmentation.

I investigated cancer susceptibility and regenerative capability of animals that are documented as fissiparous or budding. If I could not find relevant information for underexplored species, — symbol was marked. It should be noted that modes of reproduction and cancer prevalence are not fully elucidated for animals listed in this table. The absence of reported cancer occurrence could be the outcome of the insufficient investigation. Furthermore, there could be interspecific variations within a taxon. This table is a rudimentary investigation which should be updated in the subsequent comprehensive studies. All animals in this table can reproduce sexually.

BD: Budding, FP: Fissiparity, NC: No cancer reported, PR: presumably cancer-resistant, HR: Highly regenerative

*Beroids (Beroida, Beroidae) do not perform fragmentation, and they do not possess regenerative capability[1].

Animal taxon	Modes of fragmentation	Cancer susceptibility	Regeneration capability
True jellyfish (Cnidaria, Scyphozoa)	BD, FP[2,3]	NC	HR[4]
Polypodium hydriforme (Cnidaria, Polypodiozoa)	FP[5]	—	Absent[6]
Comb jellyfish (Ctenophora)	BD, FP[1,7,8]	NC[9]	HR[1]*
Hydra	BD[10]	Susceptible[10,11]	HR[12]
Starfish (Echinodermata, Asteroidea)	FP[13,14]	NC[9]	HR[15]
Brittle star (Echinodermata, Ophiuroidea)	FP[16,17]	Susceptible[9]	HR[17]
Sponge (Porifera)	BD, FP[18,19]	NC, PR[20]	HR[21]
Annelid (Annelida)	FP[22–24]	NC	HR[25]
Sea anemone (Cnidaria, Anthozoa, Actiniaria)	BD, FP[26,27]	NC	HR[27,28]
Sea cucumber (Echinodermata, Holothuroidea)	FP[29,30]	Disputable[9,31]	HR[32]
Acorn worm (Hemichordata, Enteropneusta)	FP[33]	NC[9]	HR[34,35]
Placozoa	BD, FP[36]	NC, PR[37]	HR[38,39]
Planaria (Platyhelminthes, Tricladida)	FP[40,41]	Susceptible[9]	HR[42]
Coral	BD, FP[43]	Susceptible[44]	HR[45]
Acoel (Acoela, Acoelomorpha)	BD, FP[46,47]	NC	HR[48,49]
Sea squirt (Ascidiacea)	BD, FP[50]	—	HR[50]
Moss animal (Bryozoa)	BD, FP[51,52]	—	—

Table S2. The parameter values used in the models.

ω_0 is the baseline fitness in Equation [15]; b_0 is revealed in Equation [22]; c_0 is the coefficient for cost of cancer-suppression capability $g(I) = c_0 I^2$.

Parameter(s)	FRPG-FR	FRPG-UD	FRAC-FR	FRAC-UD	BINF-FRa	BINF-FRb	BINF-UD
f_1, f_2, f_3	5, 1, 0.5	2, 1, 0.5	3, 2.5, 1	3, 2.5, 1	1, 1, 1	1, 1, 1	1, 1, 1
r_1, r_2, r_3	1.2, 1, 0.05	5, 0.5, 0.01	2, 1, 0.05	2, 1, 0.05	1.2, 1, 0.05	1.2, 1, 0.05	1.2, 1, 0.05
Δ_V, Δ_W	0.7, 0.5	0.95, 0.9	0.5, 0.3	0.9, 0.8	0.5, 0.3	0.7, 0.5	0.9, 0.8
ω_0	0.5	0.5	2	0.5	2	2	0.5
τ, δ	0.5, 3	0.3, 0.1	2, 2	0.5, 0.3	2, 3	0.5, 3	0.05, 0.1
b_0	0.5	0.1	0.5	0.2	0.7	0.5	0.1
T_U, T_F	35, 15	35, 15	25, 20	25, 20	20, 5	20, 5	20, 10
$\alpha_N, \alpha_D, \alpha_C$	7, 5, 0	7, 5, 0	7, 5, 0	7, 5, 0	7, 5, 0	3, 2.8, 0	7, 5, 0
ε	2	1.1	2	2	2	1	2
c_0	5	5	5	5	6	2	5
Offspring number	1	1	1	1	2	2	2

References

1. Martindale, M. Q. The onset of regenerative properties in ctenophores. *Curr Opin Genet Dev* **40**, 113–119 (2016).
2. Arai, M. N. *A Functional Biology of Scyphozoa*. (Springer Science & Business Media, 1997).
3. Schiariti, A. *et al.* Asexual reproduction strategies and blooming potential in Scyphozoa. *Mar Ecol Prog Ser* **510**, 241–253 (2014).
4. Sinigaglia, C. *et al.* Pattern regulation in a regenerating jellyfish. *Elife* **9**, e54868 (2020).
5. Raikova, E. V. Life cycle, cytology, and morphology of *Polypodium hydriforme*, a coelenterate parasite of the eggs of acipenseriform fishes. *J Parasitol* 1–22 (1994).
6. Raikova, E. V. Cytomorphological peculiarities of *Polypodium hydriforme* (Cnidaria). *Journal of the Marine Biological Association of the United Kingdom* **88**, 1695–1702 (2008).
7. Pang, K. & Martindale, M. Q. Comb jellies (Ctenophora): a model for basal metazoan evolution and development. *Cold Spring Harb Protoc* **2008**, pdb-emo106 (2008).
8. Freeman, G. Studies on regeneration in the creeping ctenophore, *Vallicula multiformis*. *J Morphol* **123**, 71–83 (1967).
9. Aktipis, A. C. *et al.* Cancer across the tree of life: Cooperation and cheating in multicellularity. *Philosophical Transactions of the Royal Society B: Biological Sciences* **370**, (2015).
10. Domazet-Lošo, T. *et al.* Naturally occurring tumours in the basal metazoan *Hydra*. *Nat Commun* **5**, 1–8 (2014).
11. Boutry, J. *et al.* Spontaneously occurring tumors in different wild-derived strains of hydra. *Sci Rep* **13**, (2023).
12. Reddy, P. C., Gungi, A. & Unni, M. Cellular and Molecular Mechanisms of *Hydra* Regeneration. in *Evo-Devo: Non-model Species in Cell and Developmental Biology* 259–290 (Springer Verlag, 2019).
13. Alves, S. L. S., Pereira, A. D. & Ventura, C. R. R. Sexual and asexual reproduction of *Coscinasterias tenuispina* (Echinodermata: Asteroidea) from Rio de Janeiro, Brazil. *Mar Biol* **140**, 95–101 (2002).
14. Barker, M. F. & Scheibling, R. E. Rates of fission, somatic growth and gonadal development of a fissiparous sea star, *Allostichaster insignis*, in New Zealand. *Mar Biol* **153**, 815–824 (2008).
15. Ben Khadra, Y. *et al.* An integrated view of asteroid regeneration: tissues, cells and molecules. *Cell Tissue Res* **370**, 13–28 (2017).
16. McGovern, T. M. Patterns of sexual and asexual reproduction in the brittle star *Ophiactis savignyi* in the Florida Keys. *Mar Ecol Prog Ser* **230**, 119–126 (2002).
17. Biressi, A. C. M. *et al.* Wound healing and arm regeneration in *Ophioderma longicaudum* and *Amphiura filiformis* (Ophiuroidea, Echinodermata): Comparative morphogenesis and histogenesis. *Zoomorphology* **129**, 1–19 (2010).
18. Wulff, J. L. Asexual fragmentation, genotype success, and population dynamics of erect branching sponges. *Mar. Biol. Ecol* **149**, 227–247 (1991).
19. Zilberberg, C., Solé-Cava, A. M. & Klautau, M. The extent of asexual reproduction in sponges of the genus *Chondrilla* (Demospongiae: Chondrosida) from the Caribbean and the Brazilian coasts. *J Exp Mar Biol Ecol* **336**, 211–220 (2006).

20. Fortunato, A. *et al. Tethya wilhelma* (Porifera) Is Highly Resistant to Radiation Exposure and Possibly Cancer. *Biology* **14**, 171 (2025).
21. Thoms, C., Hentschel, U., Schmitt, S. & Schupp, P. J. Rapid tissue reduction and recovery in the sponge *Aplysinella* sp. *Mar Biol* **156**, 141–153 (2008).
22. Yoshida-Noro, C. & Tochinai, S. Stem cell system in asexual and sexual reproduction of *Enchytraeus japonensis* (Oligochaeta, Annelida). *Dev Growth Differ* **52**, 43–55 (2010).
23. Oliver, J. S. Selection for asexual reproduction in an Antarctic polychaete worm. *Marine ecology progress series. Oldendor* **19**, 33–38 (1984).
24. Zattara, E. E. & Bely, A. E. Phylogenetic distribution of regeneration and asexual reproduction in Annelida: regeneration is ancestral and fission evolves in regenerative clades. *Invertebrate Biology* **135**, 400–414 (2016).
25. Özpolat, B. D. & Bely, A. E. Developmental and molecular biology of annelid regeneration: a comparative review of recent studies. *Curr Opin Genet Dev* **40**, 144–153 (2016).
26. Bocharova, E. S. & Kozevich, I. A. Modes of reproduction in sea anemones (Cnidaria, Anthozoa). *Biology Bulletin* **38**, 849–860 (2011).
27. Geller, J. B., Fitzgerald, L. J. & King, C. E. Fission in sea anemones: integrative studies of life cycle evolution. *Integr Comp Biol* **45**, 615–622 (2005).
28. Miramón-Puértolas, P., Pascual-Carreras, E. & Steinmetz, P. R. H. A population of Vasa2 and Piwi1 expressing cells generates germ cells and neurons in a sea anemone. *Nat Commun* **15**, 8765 (2024).
29. Uthicke, S. The process of asexual reproduction by transverse fission in *Stichopus chloronotus* (greenfish). *SPC Beche-de-Mer Information Bulletin* **14**, 23–25 (2001).
30. Purwati, P. Fissiparity in *Holothuria leucospilota* from tropical Darwin waters, northern Australia. *SPC Beche-de-mer Information Bulletin* **20**, 26–33 (2004).
31. Smith, A. C. & Saba, F. Comparative Pathology Human Disease Counterparts in Marine Animals. *Arch Pathol Lab Med* **124**, 348–352 (2000).
32. Zhang, X. *et al.* The sea cucumber genome provides insights into morphological evolution and visceral regeneration. *PLoS Biol* **15**, (2017).
33. Miyamoto, N. & Saito, Y. Morphological characterization of the asexual reproduction in the acorn worm *Balanoglossus simodensis*. *Dev Growth Differ* **52**, 615–627 (2010).
34. Yoshimura, K., Morino, Y. & Wada, H. Regeneration of the acorn worm pygochord with the implication for its convergent evolution with the notochord. *Dev Growth Differ* **61**, 158–165 (2019).
35. Arimoto, A. & Tagawa, K. Regeneration in the enteropneust hemichordate, *Ptychodera flava*, and its evolutionary implications. *Dev Growth Differ* **60**, 400–408 (2018).
36. Eitel, M., Guidi, L., Hadrys, H., Balsamo, M. & Schierwater, B. New insights into placozoan sexual reproduction and development. *PLoS One* **6**, (2011).
37. Fortunato, A., Fleming, A., Aktipis, A. & Maley, C. C. Upregulation of DNA repair genes and cell extrusion underpin the remarkable radiation resistance of *Trichoplax adhaerens*. *PLoS Biol* **19**, (2021).
38. Romanova, D. Y., Nikitin, M. A., Shchenkov, S. V. & Moroz, L. L. Expanding of Life Strategies in Placozoa: Insights From Long-Term Culturing of *Trichoplax* and *Hoilungia*. *Front Cell Dev Biol* **10**, (2022).

39. Mayorova, T. D. *et al.* Placozoan fiber cells: mediators of innate immunity and participants in wound healing. *Sci Rep* **11**, (2021).
40. Hoshi, M., Kobayashi, K., Arioka, S., Hase, S. & Matsumoto, M. Switch from Asexual to Sexual Reproduction in the Planarian *Dugesia ryukyuensis*. *Integr Comp Biol* **43**, 242–246 (2003).
41. Zayas, R. M. *et al.* The planarian *Schmidtea mediterranea* as a model for epigenetic germ cell specification: Analysis of ESTs from the hermaphroditic strain. *Proceedings of the National Academy of Sciences* **102**, 18491–18496 (2005).
42. Rink, J. C. Stem cell systems and regeneration in planaria. *Dev Genes Evol* **223**, 67–84 (2013).
43. Ayre, D. & Connell, J. H. The evolutionary ecology of corals. *Trends Ecol Evol* **7**, 292–295 (1992).
44. Peters, E. C., Halas, J. C. & Mccarty3, H. B. Calicoblastic neoplasms in *Acropora palmata*, with a review of reports on anomalies of growth and form in corals. *J Natl Cancer Inst* **76**, 895–912 (1986).
45. Toh, T. C. & Ng, C. S. L. Tentacular autotomy and polyp regeneration in the scleractinian coral *Euphyllia glabrescens*. *Coral Reefs* **35**, 819 (2016).
46. Fischer Verlag, G., Uirn, L., Bartolomaeus, T. & vonne Balzer, I. *Convolutriloba longifissura*, nov. spec. (Acoela)—first case of longitudinal fission in Plathelminthes. *Microfauna Marina* **11**, 7–18 (1997).
47. Shannon Iii, T. & Achatz, J. G. *Convolutriloba macropyga* sp. nov., an uncommonly fecund acoel (Acoelomorpha) discovered in tropical aquaria. *Zootaxa* **1525**, 1–17 (2007).
48. Raz, A. A., Srivastava, M., Salvamoser, R. & Reddien, P. W. Acoel regeneration mechanisms indicate an ancient role for muscle in regenerative patterning. *Nat Commun* **8**, (2017).
49. Srivastava, M., Mazza-Curll, K. L., Van Wolfswinkel, J. C. & Reddien, P. W. Whole-body acoel regeneration is controlled by Wnt and Bmp-Admp signaling. *Current Biology* **24**, 1107–1113 (2014).
50. Kürn, U., Rendulic, S., Tiozzo, S. & Lauzon, R. J. Asexual propagation and regeneration in colonial ascidians. *Biol Bull* **221**, 43–61 (2011).
51. Schwaha, T., Handschuh, S., Redl, E. & Walzl, M. G. Organogenesis in the budding process of the freshwater bryozoan *Cristatella mucedo* Cuvier, 1798 (bryozoa, phylactolaemata). *J Morphol* **272**, 320–341 (2011).
52. Hakansson, E. & Thomsen, E. Asexual propagation in cheilostome Bryozoa: evolutionary trends in a major group of colonial animals. in *Evolutionary patterns: growth, form and tempo in the fossil record (eds JBC Jackson, S. Lidgard & FK McKinney)* 326–347 (2001).
