# Supplementary figures and images for: Vertical Cancer Transmission via Asexual Fragmentation and Associated Cancer Prevalence
Source: Evol Appl. 2025 May 21;18(5):e70111. doi: 10.1111/eva.70111 (PMC12093053; doi:10.1111/eva.70111)

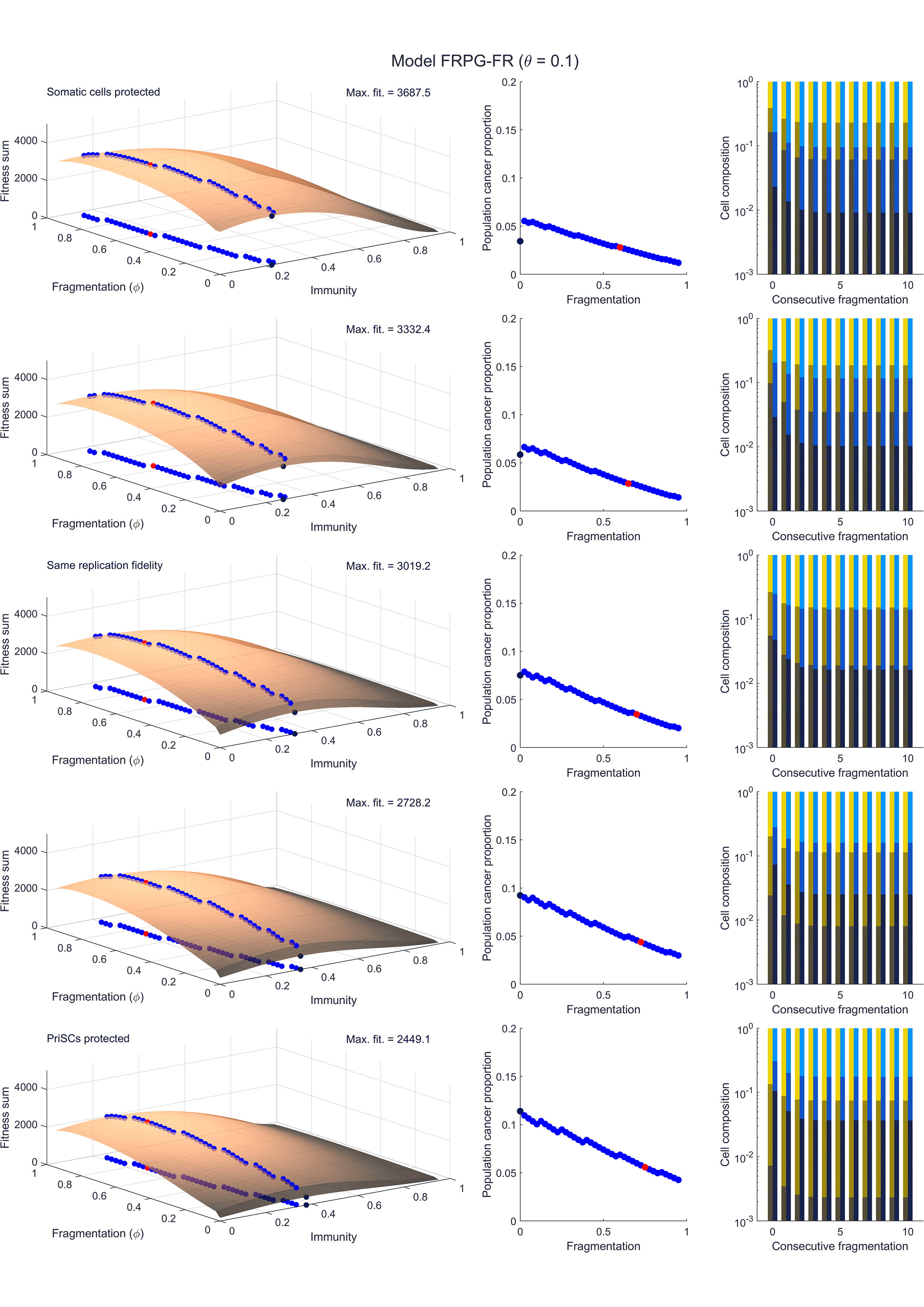

Supplement: Supplementary file 2 — Video S1. Results of FRPG‐FR model. [file EVA-18-e70111-s008.gif]

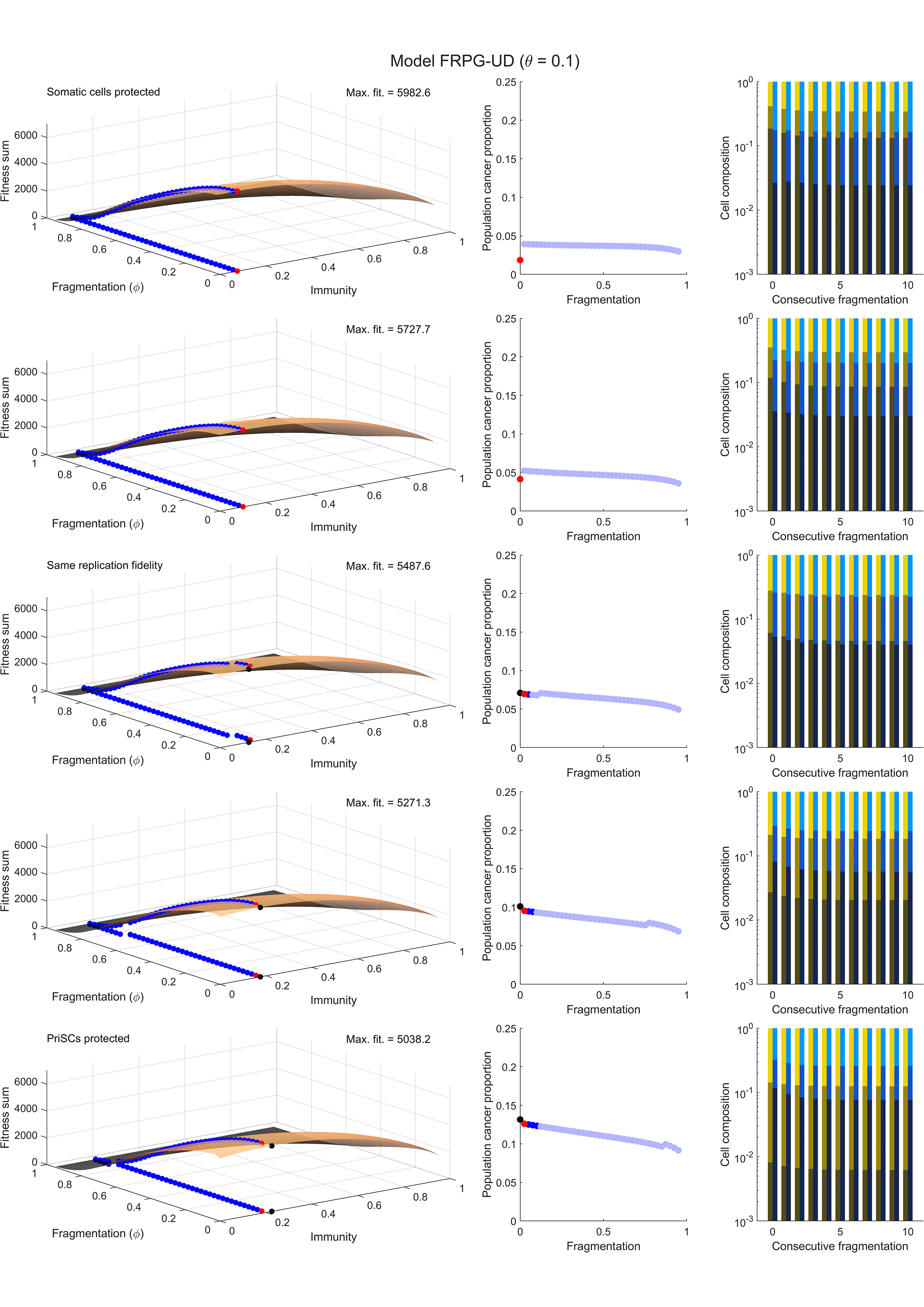

Supplement: Supplementary file 3 — Video S2. Results of FRPG‐UD model. [file EVA-18-e70111-s005.gif]

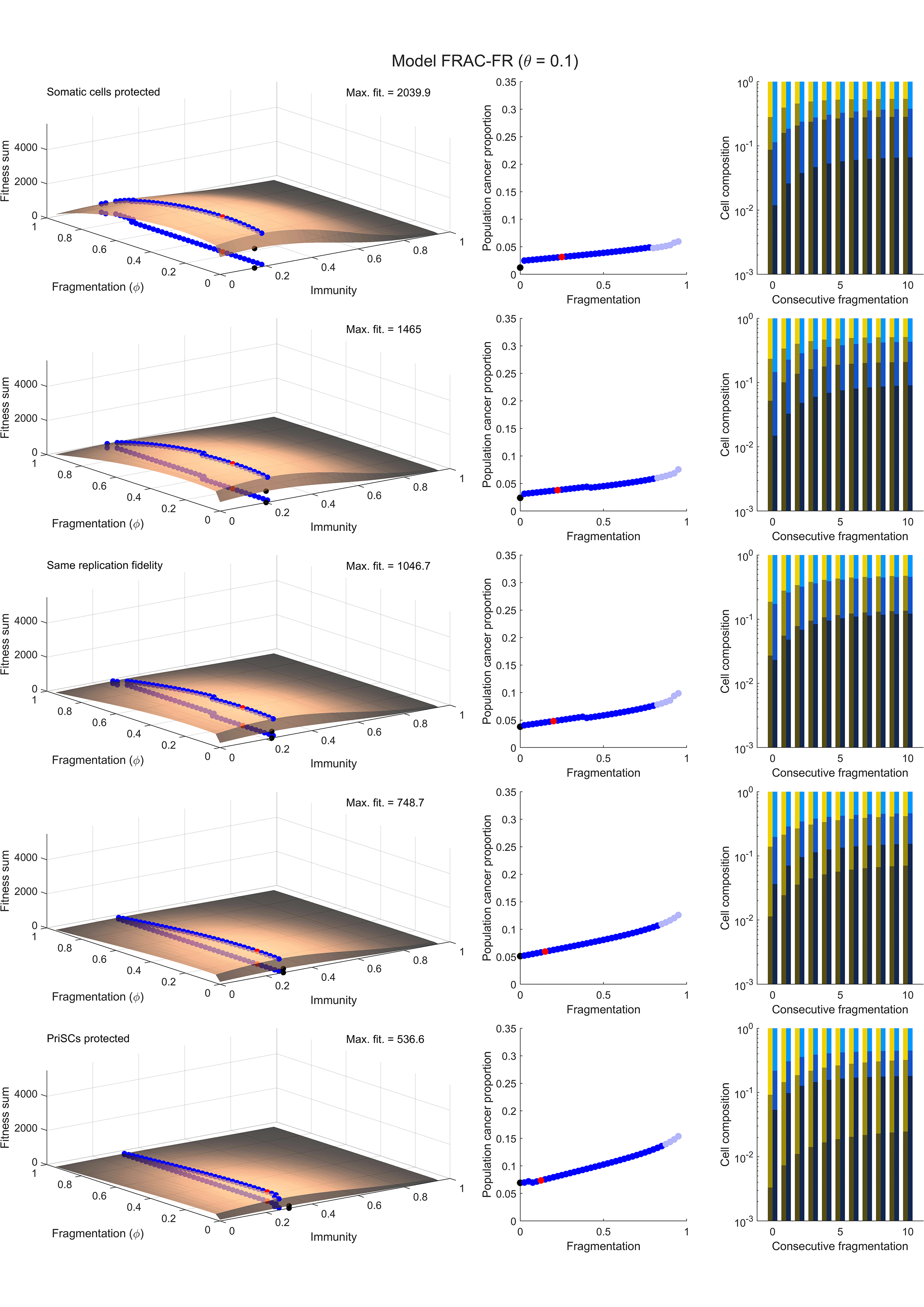

Supplement: Supplementary file 4 — Video S3. Results of FRAC‐FR model. [file EVA-18-e70111-s006.gif]

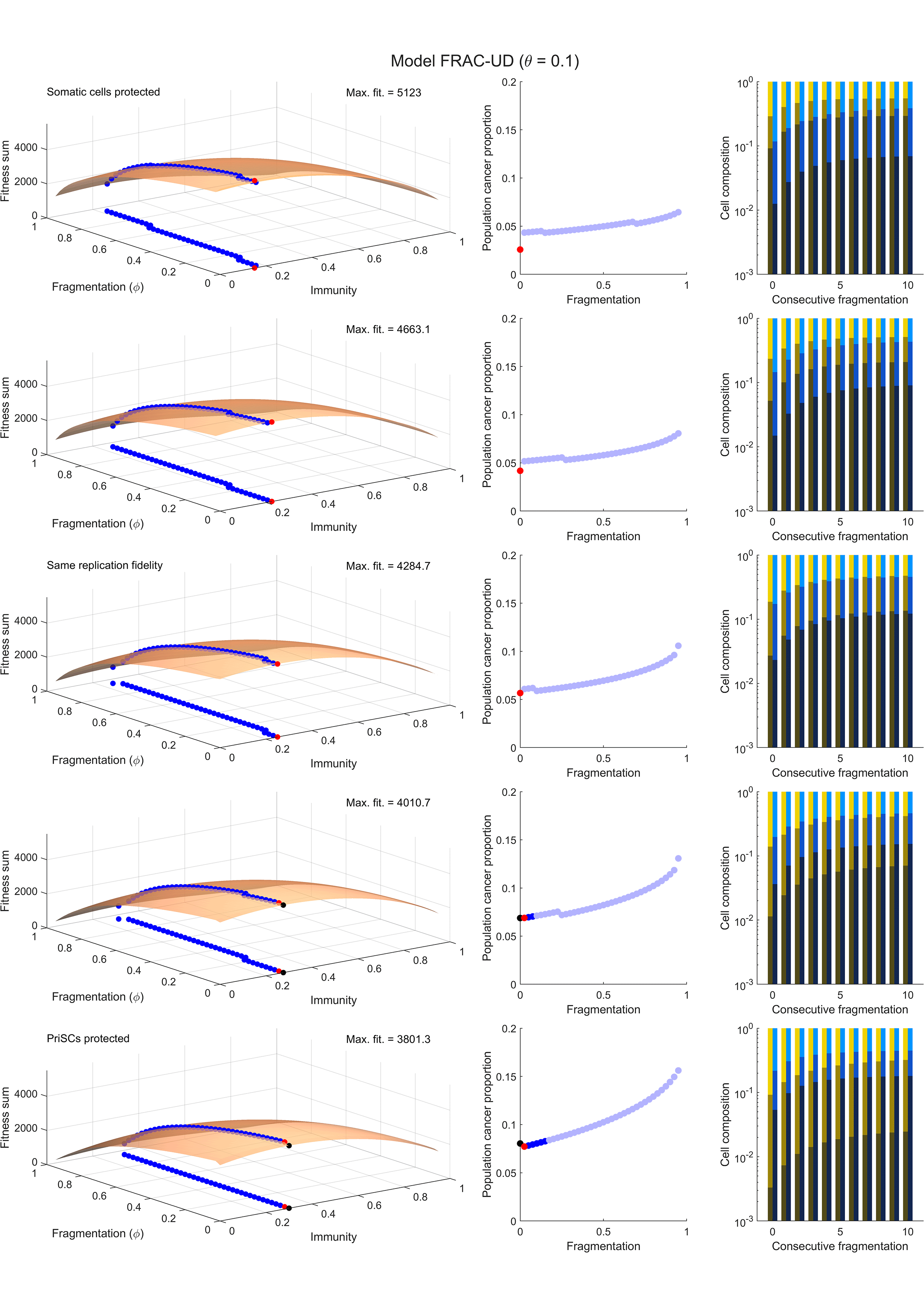

Supplement: Supplementary file 5 — Video S4. Results of FRAC‐UD model. [file EVA-18-e70111-s003.gif]

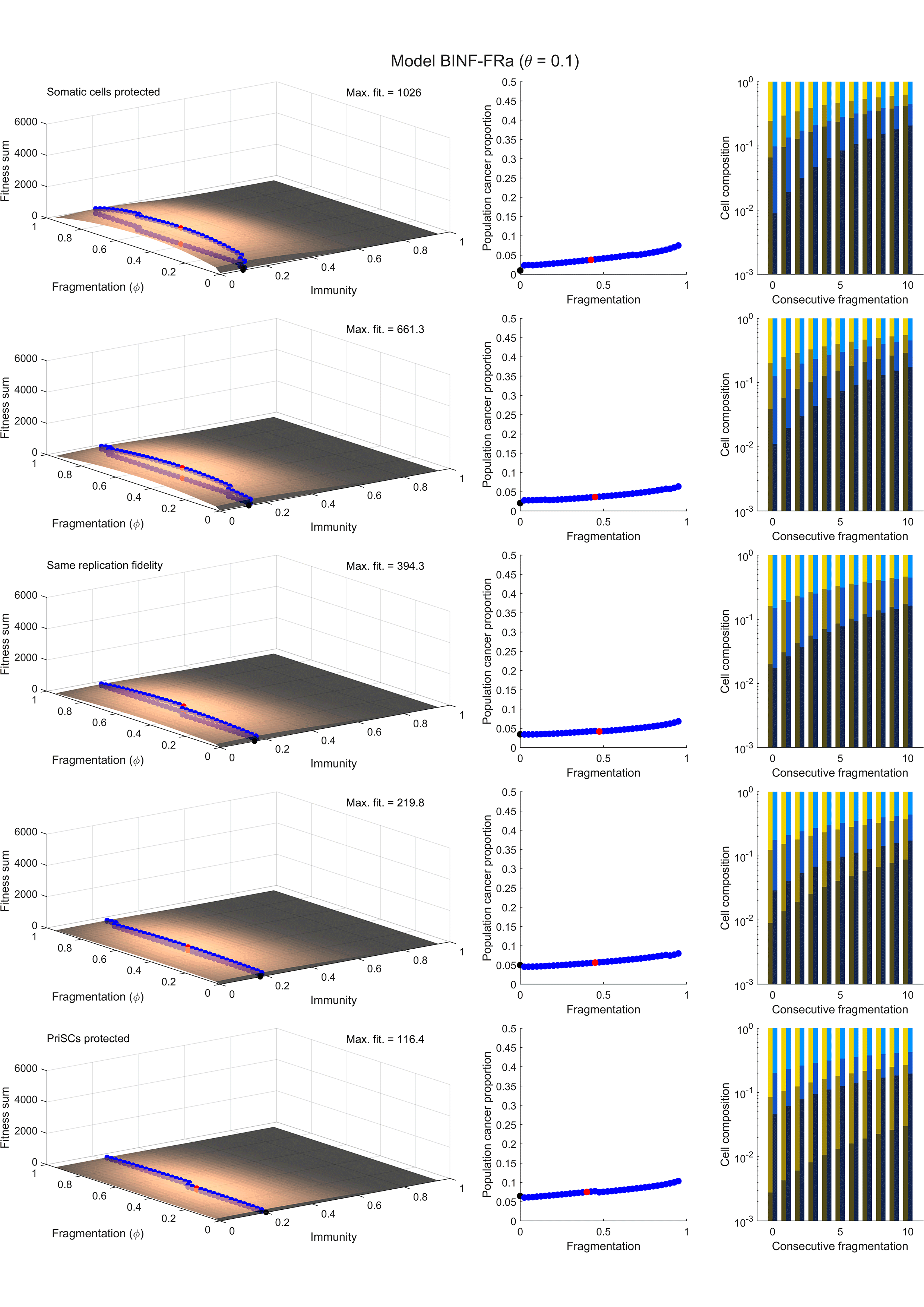

Supplement: Supplementary file 6 — Video S5. Results of BINF‐FRa model. [file EVA-18-e70111-s007.gif]

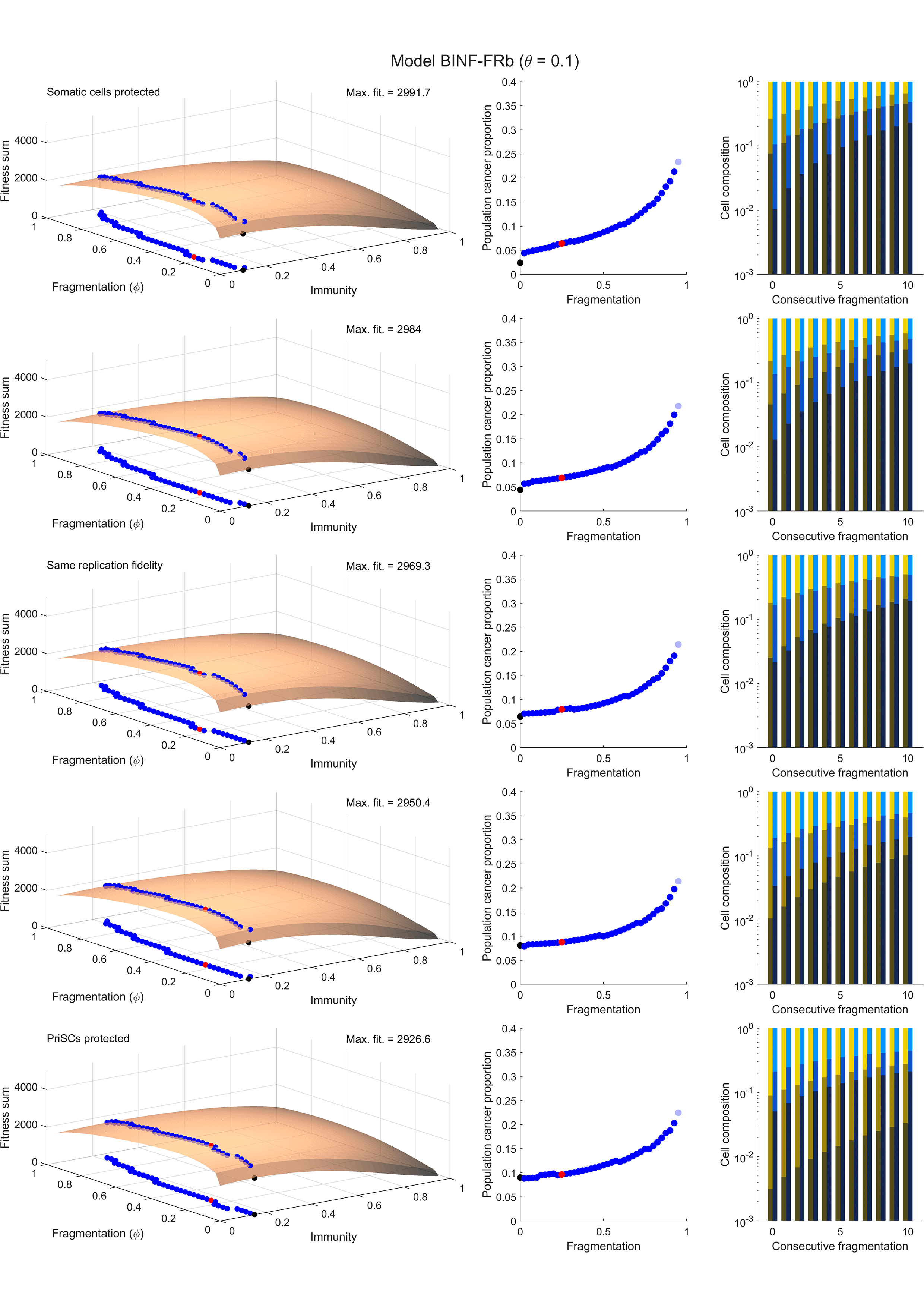

Supplement: Supplementary file 7 — Video S6. Results of BINF‐FRb model. [file EVA-18-e70111-s002.gif]

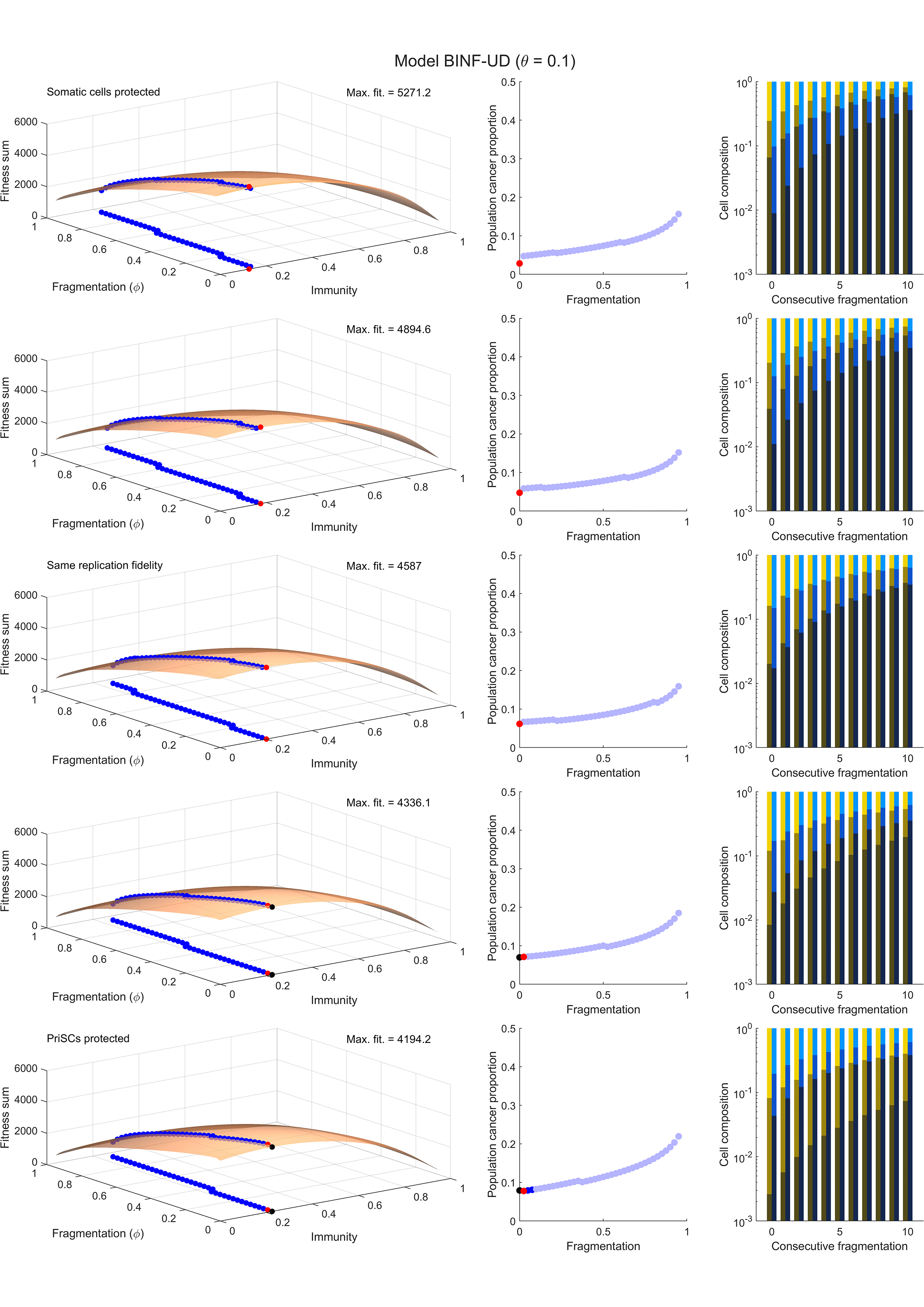

Supplement: Supplementary file 8 — Video S7. Results of BINF‐UD model. [file EVA-18-e70111-s004.gif]
